# Supplementary material for: Ancient genomes in South Patagonia reveal population movements associated with technological shifts and geography
Source: Nat Commun. 2020 Aug 3;11:3868. doi: 10.1038/s41467-020-17656-w (PMC7400565; doi:10.1038/s41467-020-17656-w)
Supplement: Supplementary file 1 — Supplementary Information [file 41467_2020_17656_MOESM1_ESM.pdf]

Ancient genomes in South Patagonia reveal population movements associated with technological shifts and geography

Nakatsuka *et al.*

Supplementary Figures

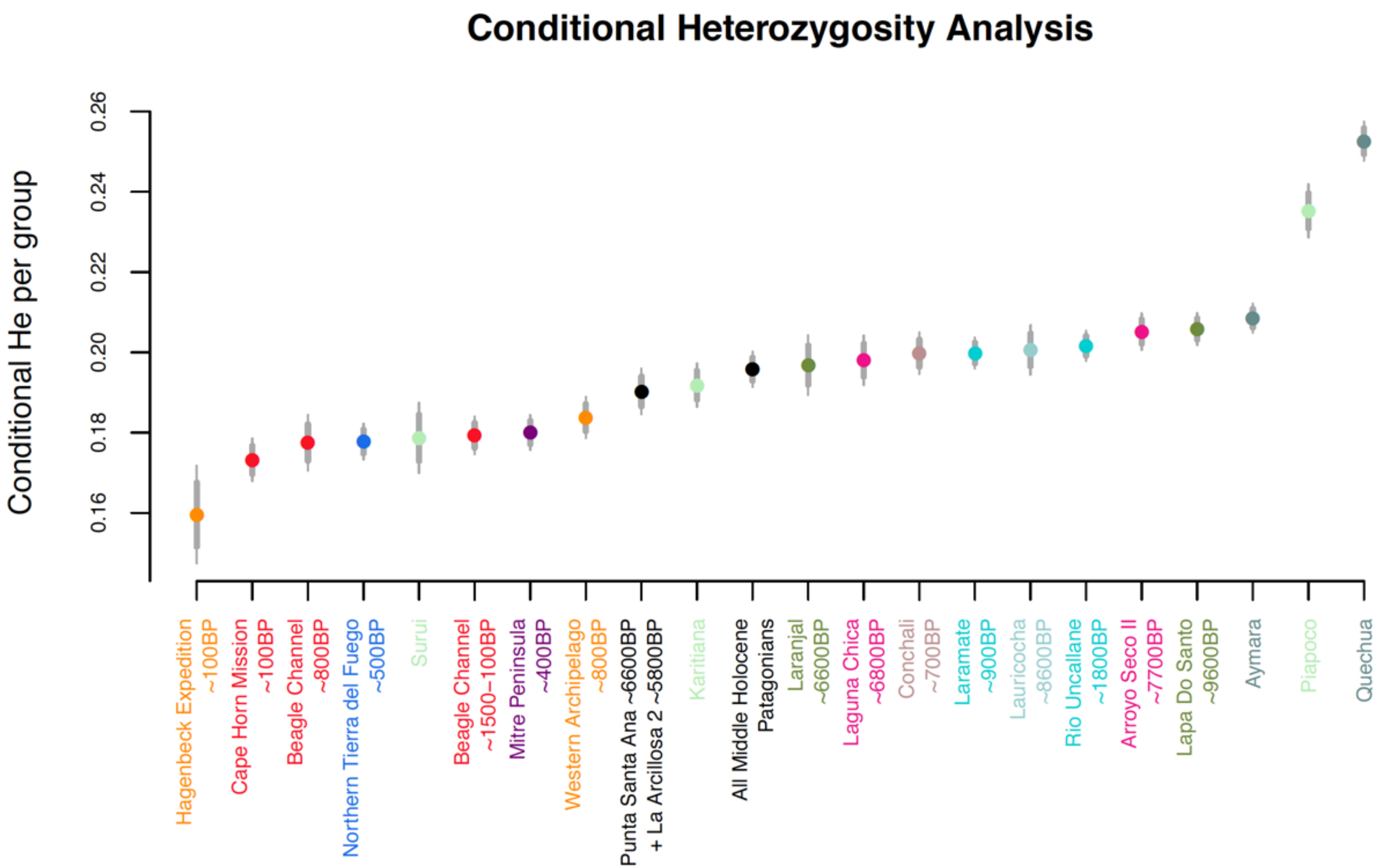

Supplementary Figure 1. Conditional heterozygosity analysis. All Middle Holocene Patagonians refers to the grouping of *Chile_PuntaSantaAna_6600BP*, *Argentina_LaArcillosa2_5800BP*, and *Chile_Ayayema_4700BP*. Narrow and thick grey segments: ± 1.96 and 3 standard errors, respectively.

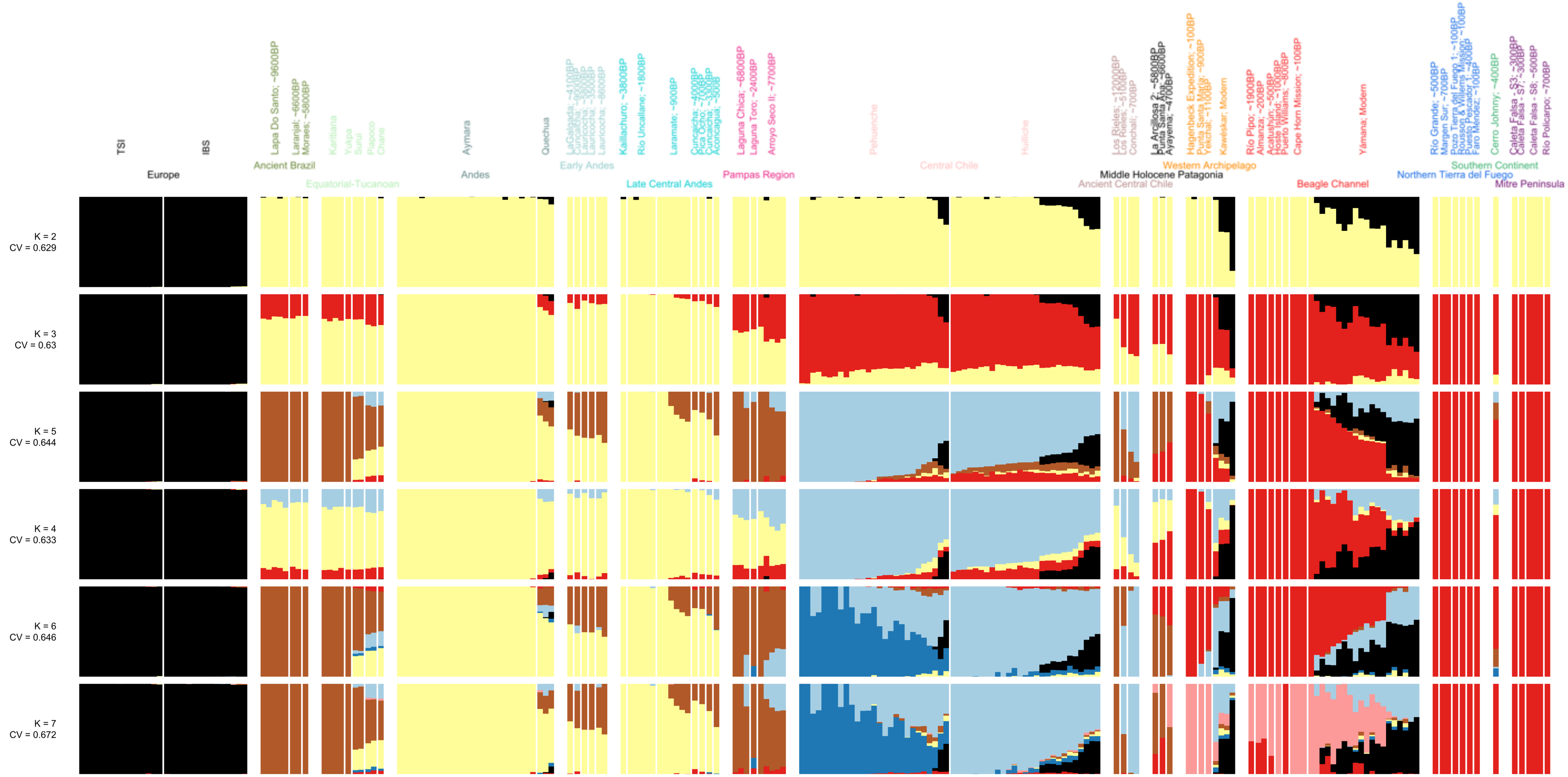

Supplementary Figure 2. Unsupervised ADMIXTURE plot at different K values from 2-7. The run with the highest log likelihood score after 10 trials was taken for each K value. The cross-validation scores are given on the left.

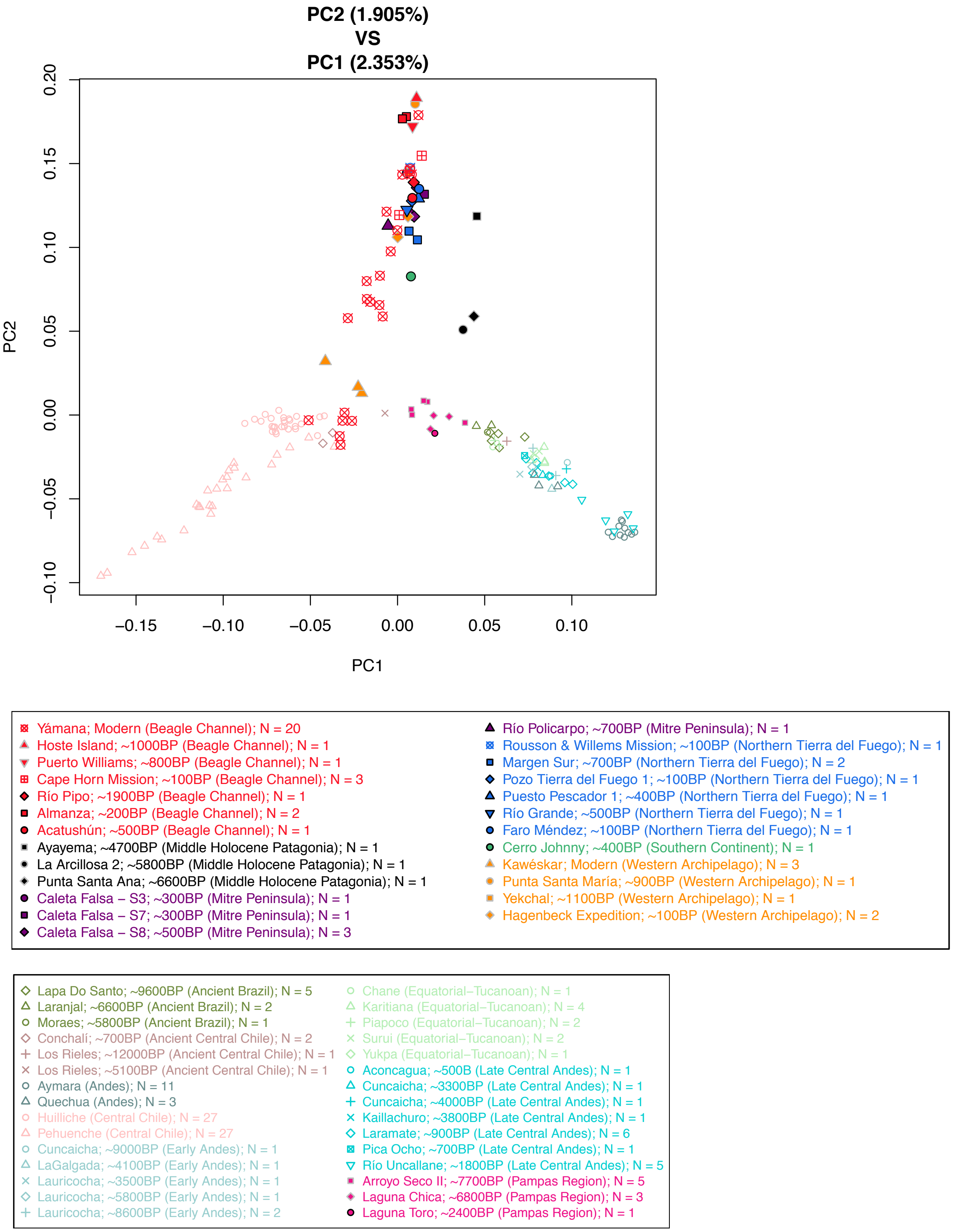

Supplementary Figure 3. Principal Components Analysis (PCA). Ancient individuals were projected onto principal components inferred from the modern individuals after masking modern individuals to remove non-Native American ancestry (see Online Methods).

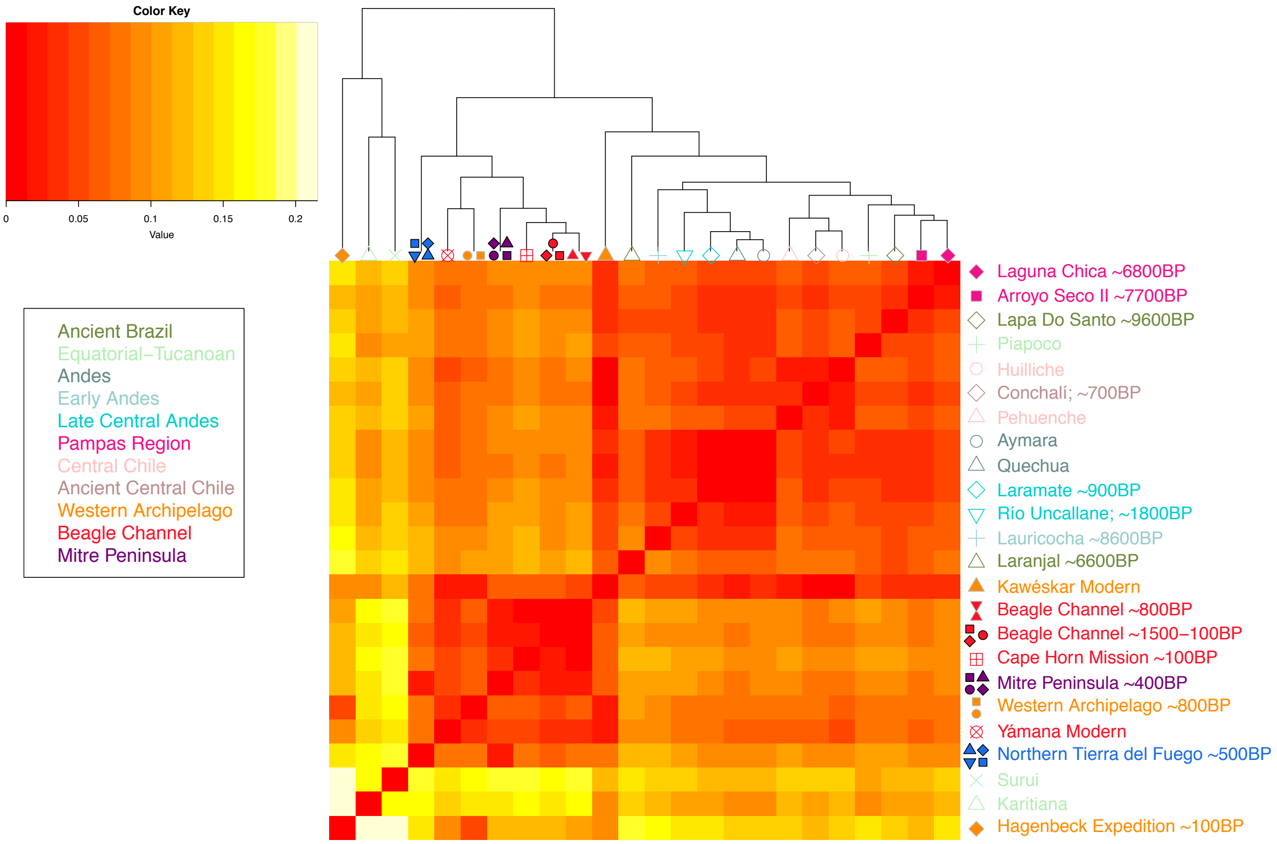

Supplementary Figure 4. Heatmap of pairwise F_{ST}. F_{ST} was estimated among South American modern and ancient populations. The dendrogram was estimated with hierarchical clustering using F_{ST} as distances (see also Supplementary Data File 3K). For point legend, refer to Supplementary Figure 3.

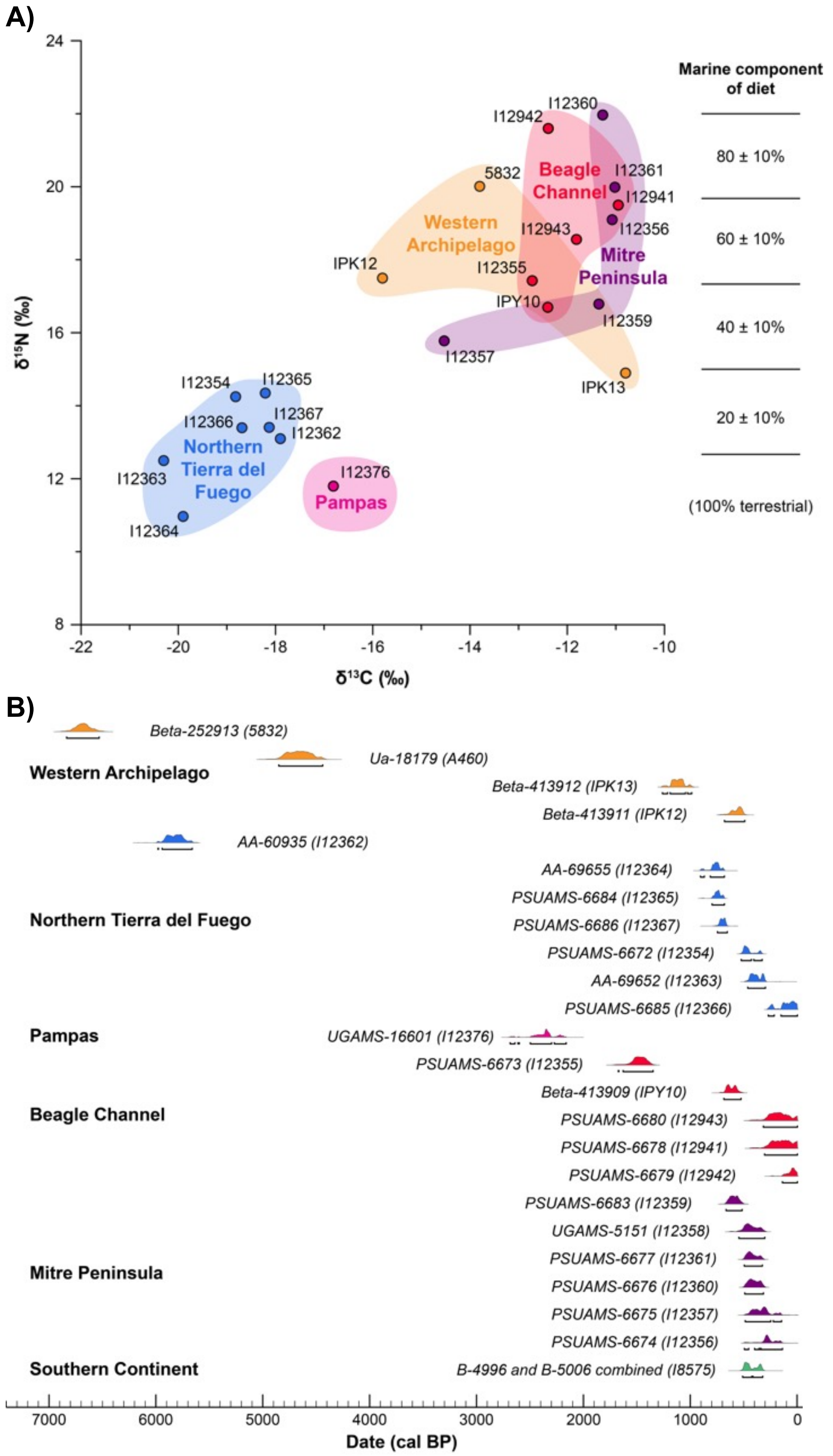

Supplementary Figure 5. Carbon and Nitrogen isotopes analyses (A) Carbon and nitrogen isotope values for all ancient individuals. When different measurements were available they were averaged (Supplementary Data File 1 contains all values). **(B)** Maritime reservoir-corrected radiocarbon date distributions of all individuals with radiocarbon dates. Bracket below each probability distribution: 2σ confidence interval.

A)

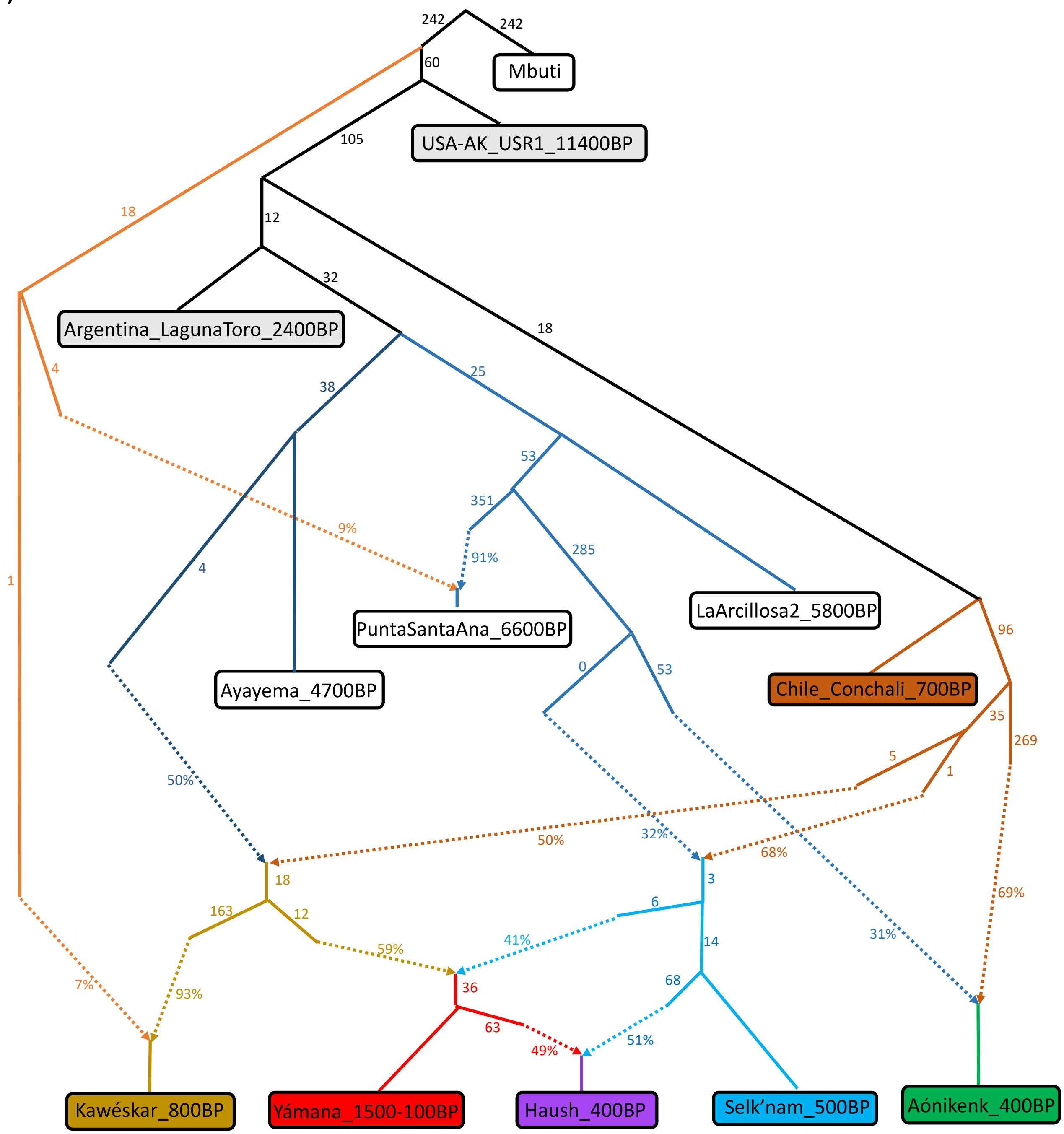
Mbuti
USA-AK_USR1_11400BP
Argentina_LagunaToro_2400BP
PuntaSantaAna_6600BP
LaArcillosa2_5800BP
Ayayema_4700BP
Chile_Conchali_700BP
Kawéskar_800BP
Yámana_1500-100BP
Haush_400BP
Selk'nam_500BP
Aónikenk_400BP
242
242
60
105
18
12
32
18
4
38
25
53
351
9%
91%
285
1
4
0
53
96
35
269
5
1
50%
50%
32%
68%
3
18
163
12
6
14
41%
59%
69%
31%
7%
93%
36
68
63
49%
51%

B)

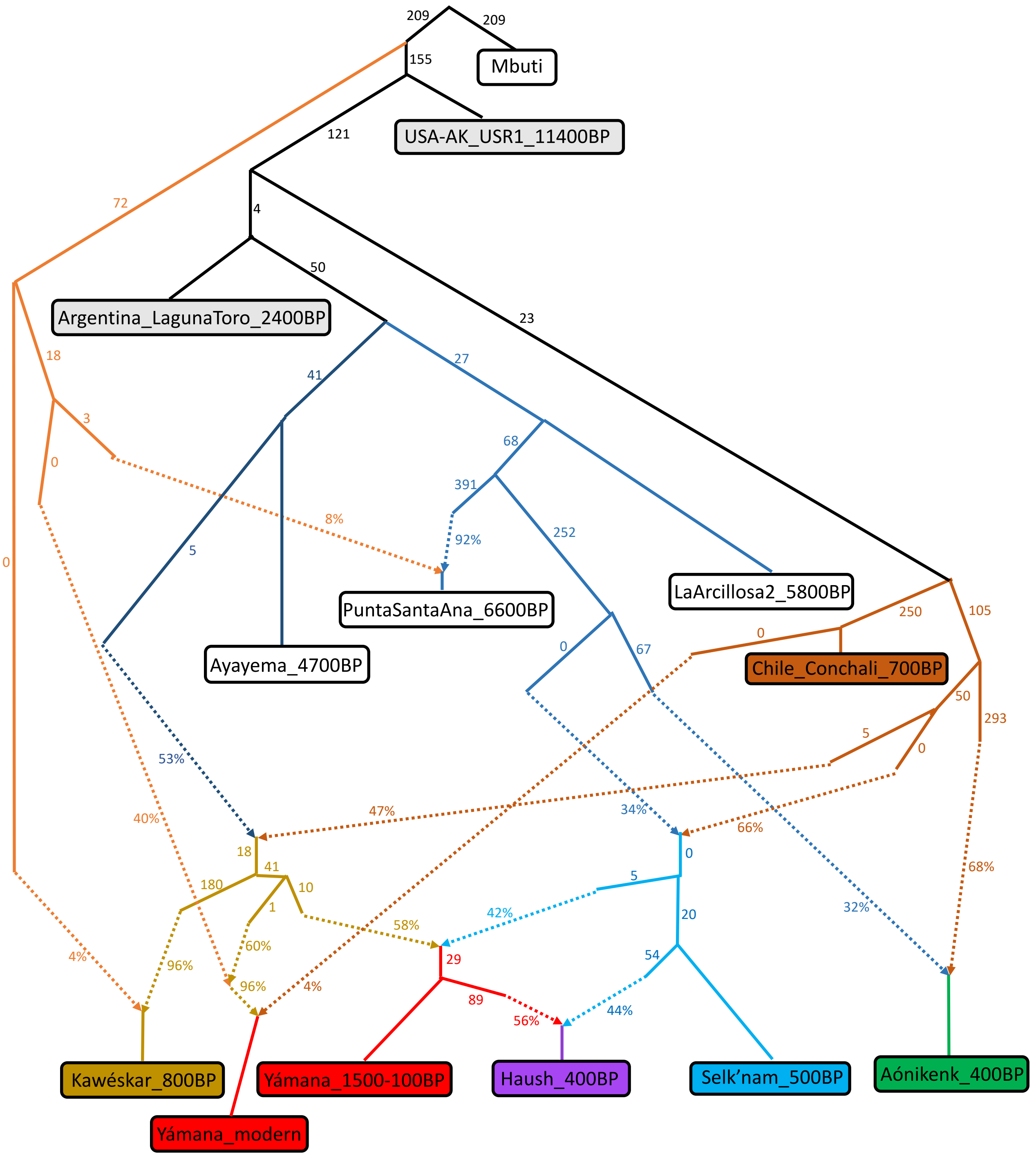

209
209
Mbuti
155
USA-AK_USR1_11400BP
121
72
4
50
23
Argentina_LagunaToro_2400BP
18
41
27
3
68
0
391
8%
92%
0
5
252
PuntaSantaAna_6600BP
LaArcillosa2_5800BP
250
105
0
0
67
Ayayema_4700BP
Chile_Conchali_700BP
50
293
5
0
53%
47%
34%
66%
40%
18
0
41
180
10
5
68%
1
42%
32%
20
58%
60%
29
54
4%
96%
96%
4%
89
56%
44%
Kawéskar_800BP
Yámana_1500-100BP
Haush_400BP
Selk'nam_500BP
Aónikenk_400BP
Yámana_modern

C)

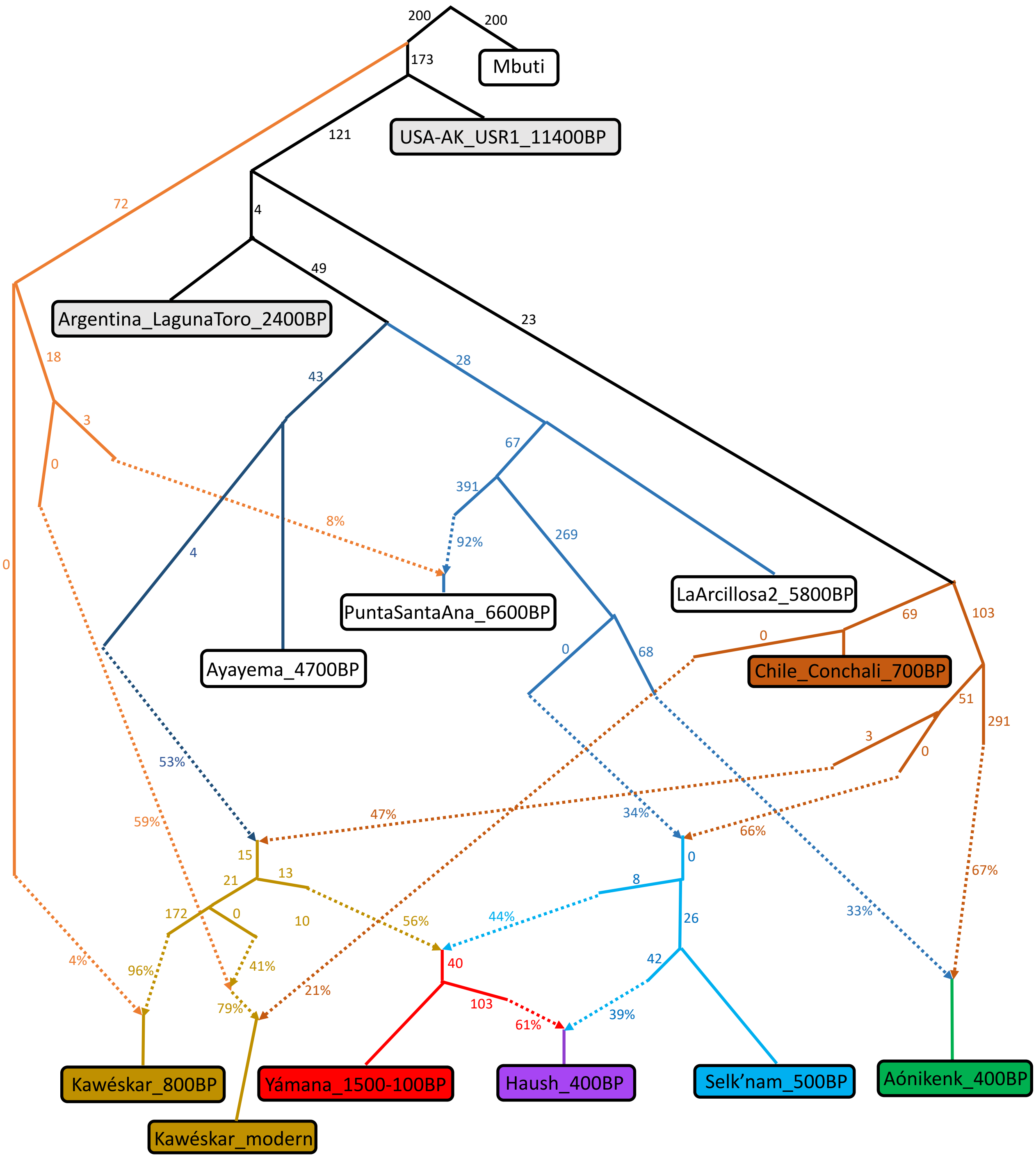
Mbuti
USA-AK_USR1_11400BP
Argentina_LagunaToro_2400BP
PuntaSantaAna_6600BP
LaArcillosa2_5800BP
Ayayema_4700BP
Chile_Conchali_700BP
Kawéskar_800BP
Yámana_1500-100BP
Haush_400BP
Selk'nam_500BP
Aónikenk_400BP
Kawéskar_modern
200
200
173
121
72
4
49
23
18
3
0
0
28
43
67
391
92%
269
8%
4
69
103
0
0
68
51
291
3
0
53%
47%
34%
66%
59%
15
13
21
172
0
10
56%
44%
8
26
33%
67%
4%
96%
41%
40
42
21%
79%
103
61%
39%

D)

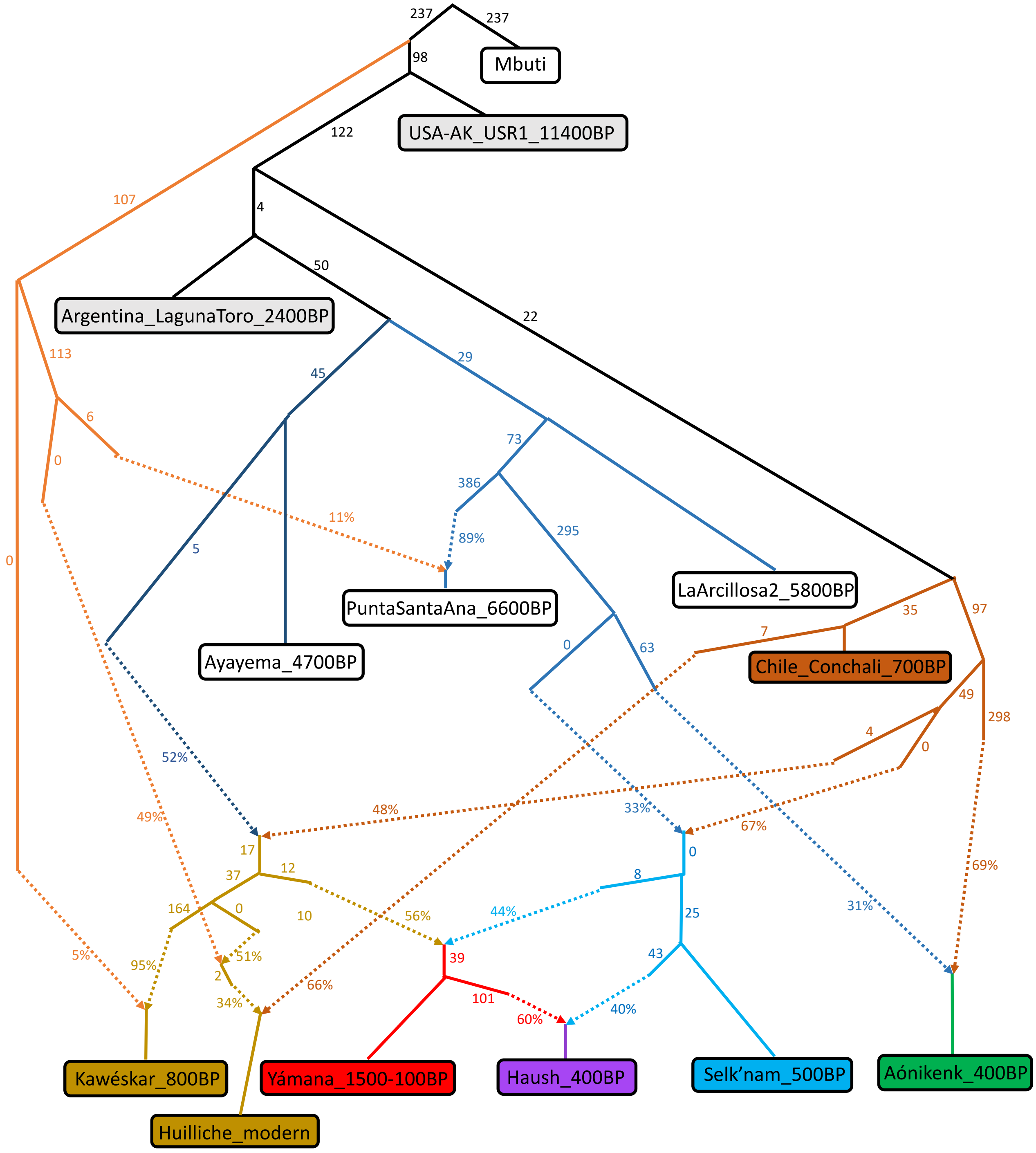
Mbuti
USA-AK_USR1_11400BP
Argentina_LagunaToro_2400BP
PuntaSantaAna_6600BP
LaArcillosa2_5800BP
Ayayema_4700BP
Chile_Conchali_700BP
Kawéskar_800BP
Yámana_1500-100BP
Haush_400BP
Selk’nam_500BP
Aónikenk_400BP
Huilliche_modern

E)

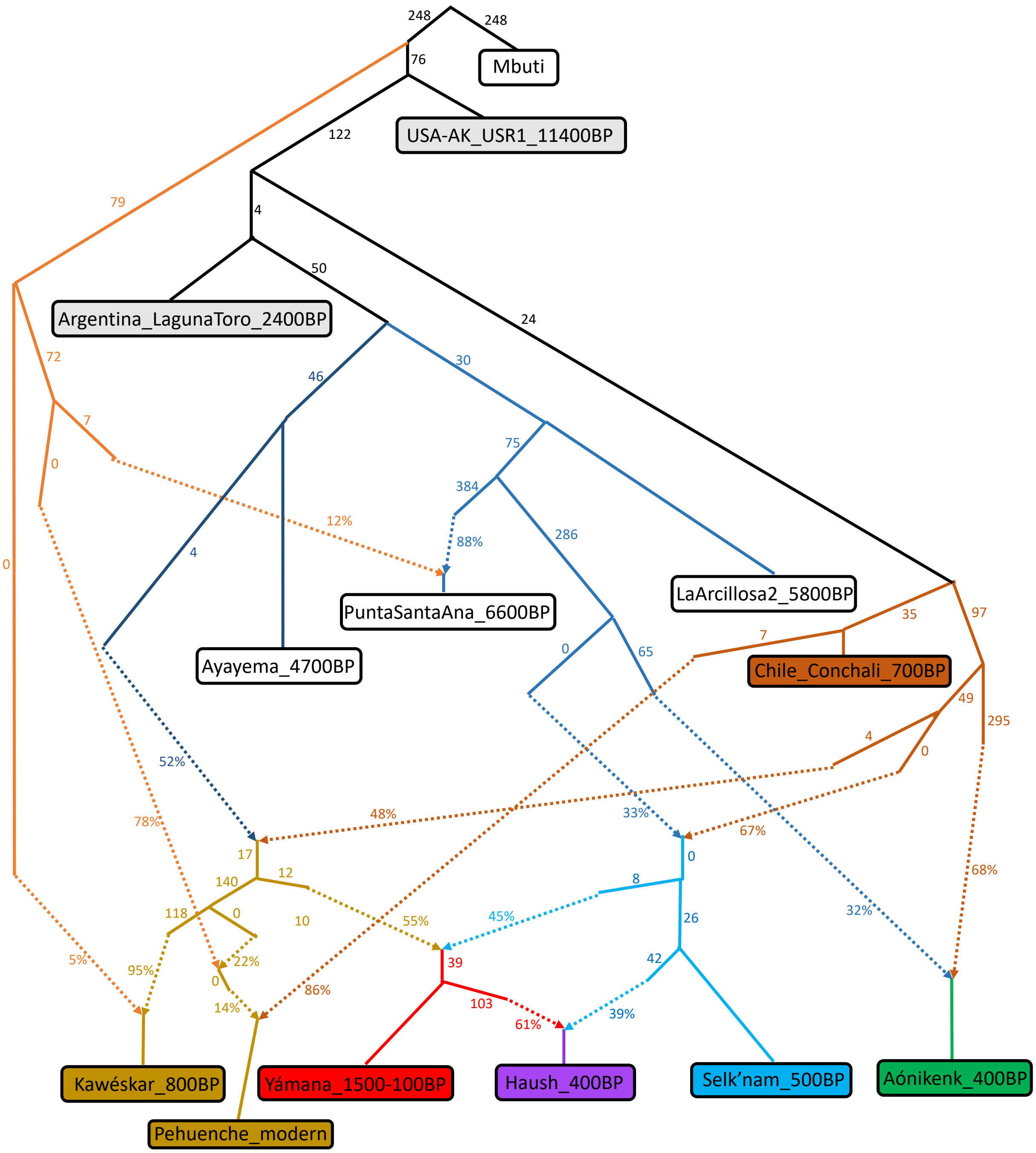

Supplementary Figure 6. Additional Admixture Graphs (A) Full admixture graph from Figure 3 with the artifactual edges into *PuntaSantaAna_6600BP* and *Kawéskar_800BP* left in. **(B-E)** Admixture graph on Axiom LAT1 unmasked genotype set with additional modern Patagonians (**(B)** Yámana, **(C)** Kawéskar, **(D)** Huilliche, and **(E)** Pehuenche) modeled in (maximum |*Z*-score| = 3.3, 3.0, 3.0, and 2.8, respectively; with transversions only the |*Z*-scores| are 3.0, 2.9, 3.3, and 3.1, respectively). The maximum |*Z*-score| is 2.8 on this SNP set without the modern Patagonians added in, and 2.8 with only transversions. We could not model the modern Patagonian groups in the same graph due to attractions of the groups with each other, which could be due to artifactual modern vs. ancient DNA bias or recent gene flow between the different groups. *Z*-scores were determined from standard errors obtained from jackknife resampling.

Supplementary Notes

Supplementary Note 1. Additional Background on Southern Patagonia:

From a material culture point of view, the shared reliance on marine resources of the two westernmost groups and on terrestrial resources of the two most northeastern groups could potentially be correlated to shared ancestry. The affinities of the Haush, whose unique food procurement strategy relying both on terrestrial resources like the Selk'nam who flank them to the north along the coast, and on marine resources like the Yámana who flank them to the west, has been of particular interest[1]. Ethnographers have documented shared symbolic systems between the northern Selk'nam and Haush—for example their joint celebration of male initiation into adulthood (the Hain)[2]—suggesting that the Haush could be viewed as an eastern subgroup of a broader entity that also includes the Selk'nam, with no similar cultural relationship to the western Yámana[3]. If the Haush are best seen as a subgroup of a larger entity also including the Selk'nam, then it would be plausible for them not to be admixed with Yámana-related ancestry.

From a linguistic point of view, there are strong correlations to diet and geography. The languages of the easternmost Aónikenk, Selk'nam, and Haush belong to the Chon family[55]. The Kawéskar and Yámana that based their subsistence primarily on marine resources are usually considered linguistic isolates, although some scholars consider their languages to be related[4,5].

From the point of view of skeletal morphology and somatometry (variation in body shapes), there are striking differences across groups. At the time of European contact, the eastern terrestrial-reliant groups had significantly larger stature (1.80 meters (m) on average) than the western maritime-reliant groups (1.57 m)[6-8]. Morphological analyses have suggested two ancestral populations, Atlantic and Pacific, consistent with the significantly different cranial size and robustness in terrestrial hunter-gatherers (Selk'nam/Haush) compared to sea canoeists (Yámana and Kawéskar)[9,10]. Some have interpreted the morphological uniformity of early skeletons from this region relative to other South Americans as evidence of a single origin in deeper time followed by differentiation within South Patagonia[11,12].

Supplementary Note 2. Details about analyzed individuals.

We generated genome-wide data from skeletal remains of 20 ancient individuals:

- Laguna Toro, Pampas, Argentina: 1
- Cerro Johnny, Southern Continent, Chile: 1
- Faro Méndez, North of Tierra del Fuego, Chile: 1
- Puesto Pescador 1, North of Tierra del Fuego, Argentina: 1
- La Arcillosa 2, North of Tierra del Fuego, Argentina: 1
- Pozo Tierra del Fuego 1, North of Tierra del Fuego, Argentina: 1
- Río Grande, North of Tierra del Fuego, Argentina: 1
- Margen Sur, North of Tierra del Fuego, Argentina: 3
- Río Policarpo, Mitre Peninsula, Argentina: 1
- Caleta Falsa, Mitre Peninsula, Argentina: 5
- Río Pipo, Beagle Channel, Argentina: 1
- Almanza, Beagle Channel, Argentina 2
- Acatushún, Beagle Channel, Argentina: 1

Many of the ancient DNA samples analyzed in this study were obtained from human teeth from individuals from Almanza (N = 2), Caleta Falsa (N = 5), Acatushún (N = 1), Río Pipo (N = 1), Río Policarpo (N = 1), and Río Grande (N = 1), currently curated in the Museo del Fin del Mundo (MFM) in Ushuaia (in the jurisdiction of the Provincial Office of Museums and Cultural Heritage - Ministry of Culture, province of Tierra del Fuego, Antarctica and South Atlantic islands, Argentina). Another group of individuals from La Arcillosa 2 (N = 1), Puesto Pescador 1 (N = 1), Pozo Tierra del Fuego 1 (N = 1), and Margen Sur (N = 3) are curated in the Centro Austral de Investigaciones Científicas (CADIC), in Ushuaia, Argentina. The individuals from Cerro Johnny (N = 1) and Faro Méndez (N = 1) are human teeth taken from the biological anthropology collection of the Universidad de Magallanes (Centro de Estudios del Hombre Austral, Instituto de la Patagonia, Punta Arenas, Chile) recognized by the Chilean National Monuments Council (CMN) as the official deposit of collections obtained in the area from 1969 to its date of foundation. This collection, the largest in the region, is the product of donations and/or archaeological research developed by the university and associated institutions. The individual from Laguna Toro (N = 1) is kept at the Universidad Nacional del Sur (Bahía Blanca, Argentina).

Anne Chapman excavated the remains from Caleta Falsa in 1969 and 1970, and one of the authors (RAG) then studied them[13]. Before her death, Dr. Chapman asked RAG to guarantee the return of the skeletons to the province of Tierra del Fuego, a request he fulfilled. The other human remains that

belong to the MFM were found by chance and/or amateur excavations during the 70s and 80s. As part of the research program led by RAG, specialized work was done to improve the conservation and storage conditions of the MFM[14], and the current digital documentary database of the MFM was also generated[15]. The individuals from the CADIC were excavated by MS and FS, who were called when human remains were found[16]. In these cases, they performed rescue archeology[17], which aims to preserve heritage that would have been lost due to environmental disturbance[18]. The individual from Laguna Toro was excavated by RJV when the remains appeared during an archaeological survey.

All Argentina samples analyzed in this work were exported with authorization from the Instituto Nacional de Antropología y Pensamiento Latinoamericano (INAPL) N° DI-2017-50-APN-INAPL#MC dated November 1st, 2017 and N° DI-2017-72-APN-INAPL#MC dated December 28th, 2017. MDDC managed the corresponding governmental permissions and extracted teeth for the purpose of performing DNA and stable isotopes analysis. These permissions were given under the understanding that the export of samples from sites from Tierra del Fuego travel with the authorization of the Provincial Office of Museums and Cultural Heritage Ministry of Culture, province of Tierra del Fuego, Antarctica and South Atlantic islands, and this procedure was followed for the present study. The export of the sample from Laguna Toro, province of Buenos Aires, had the authorization from the government's Direction of Museums and Preservation of Buenos Aires Province. Skeletal material from the 2 Chilean individuals were exported with permissions from the Consejo de Monumentos Nacionales de Chile in 2009.

Description of Archaeological Sites:

A summary of all information obtained for genetic or radiocarbon dating, including genetic sex and carbon and nitrogen isotope data is presented in Supplementary Data File 1.

Laguna Toro (Argentina): 2690-2150 BP

- I12376: 2690-2150 cal BP (2369±52 BP, UGAMS-16601)

The Laguna Toro site (38° 50' 36.45'' S and 61° 25' 12.44'' W) is located in the southwest of the province of Buenos Aires in Coronel Dorrego department. It receives its name from a lagoon situated 12 km from the Atlantic coast. At the southeast of the lagoon, a series of 9 transects of 5 m wide and 225 m long were made. The site presents an important set of lithic materials (tools, cores, flakes, debris, and nodules), archaeofaunistic remains, and ceramic pots. One sample (I12376) analyzed in this study is derived from a female adult who was found in a sector adjacent to the transects. A 2x1 m excavation

was carried out, resulting in the finding of a single individual, buried in a primary way, in a North-South orientation and deposited in a small wind-generated hollow in the sediment. Stable isotope values of δ13C(col)= –16.8‰, δ13C(ap)= –13.5, and δ15N= 11.8‰ obtained for this individual indicate a diet based predominantly on terrestrial resources[19].

Cerro Johnny (Chile): 520-320 BP

- I8575: 520-320 cal BP: From two dates of the same individual: [428±46 BP, B-4996 and B-5006 combined)]

The Cerro Johnny skeleton was accidentally found by shepherd Jovino Díaz and subsequently excavated by archaeologists Mateo Martinic and Pedro Cárdenas in 1975. The site is located in a small cave that opens on a hill dominating the lava field of Pali-Aike, in *estancia* Brazo Norte, Magallanes. The cave itself measures 1m high, 0.80m wide and ~1.5m deep. The archaeologists retrieved about 93% of the skeleton, mostly on the surface of the cave, along with some mummified tissues; the body was lying on his right side in the fetal position. Among the bones, some fragments of painted leather, maybe from a guanaco, compatible with the traditional Aónikenk *quillangos*, were found. It has been suggested that the body might have been wrapped up in a *quillango*[20].

Faro Méndez (Chile): 150-50 BP

- I8576: 150-50 BP

The Faro Méndez remains seem to belong to a rather recent Selk'nam individual, donated to the Universidad de Magallanes museum in 1997 by Mr. Ricardo Olea, who found these remains in his property in the north of Isla Grande de Tierra del Fuego. The name refers to Punta Méndez in the first Angostura of the Magallanes channel where there is a lighthouse of the same name at the geographical coordinates: 52° 31.6' S and 69° 35.014' W. It corresponds to a young adult (around 25 years old) male with part of his skeleton preserved and an incomplete skull. His teeth are heavily worn with several of them showing signals of dental abscesses. The remains themselves lack archaeological information and context, but they come from a region where Selk'nam archaeological sites are abundant, including the Punta Baxa 7 site[21,22].

<u>Puesto Pescador 1 (Argentina):</u> 460-290 BP

- I12363: 460-290 cal BP (335±35 BP, AA69652)

Puesto Pescador 1 (PP1) (53º 17' 21.605" S and 68º 28.73' W) is located at the northwest of San Sebastian Bay, northern Isla Grande de Tierra del Fuego. It was detected by chance when a machine operator uncovered a human burial and segmented it in two parts. The skeleton was deposited at about 30 cm depth. This soil is on beach ridges composed of gravels of different sizes, between 10 and 40 mm, that were assigned to the Late Holocene.

The burial reconstruction determined that the skeleton was in an extended ventral position, with the left arm stretched out along the body and the right one folded against the chest, likely with the hand closed. The skeleton is 48% complete and corresponds to a male of 21-25 years old. Stable isotope values of $\delta^{13}C(col)= -20.3‰$, and $\delta^{15}N= 12.5‰$ obtained for this individual suggest a terrestrial diet[23,24]. The presence of two projectile points made on chalcedony within the thoracic region and some blows to the skull suggests that the remains of PP1 might represent one of few cases with injuries of interpersonal violence known for the northern coast of Tierra del Fuego[24].

<u>La Arcillosa 2 (Argentina):</u> 5990-5660 BP

- I12362: 5990-5660 cal BP (5205±58 BP, AA60935)

The La Arcillosa 2 (LA2) site (53º 34.45' S 68º 02.257' W) is located approximately 2 km west of the Atlantic coast (northern Isla Grande de Tierra del Fuego). It is a shell midden containing cultural materials (lithic and faunal remains) and is included in aeolian deposits that cover a cliff 8 meters above the Chico river floodplain. This cliff marks the maximum point of the Middle Holocene marine transgression[25].

One sample (I12362) analyzed in this study comes from a primary human burial that was recovered lying under the shell midden; it was found in a lateral decubitus position. The bones belong to a female adult (21-25 years old). The skeleton was 94% complete and the bones show signs of greater physical activity in the arm than in the legs[16]. An exploratory biomechanical analysis performed on tibias shows that the LA2 skeleton is less sturdy than other individuals of the same region[26]. Stable isotope values of $\delta^{13}C(col)= -17.9‰$, and $\delta^{15}N= 13.1‰$ obtained for this individual indicate a diet based predominantly on terrestrial resources[16].

Pozo Tierra del Fuego 1 (Argentina): 280-0 BP

- I12366: 280-0 cal BP (220±20 BP, PSUAMS-6685))

One sample (I12366) analyzed in this study is derived from isolated human bones from the Pozo Tierra del Fuego 1 site (53° 39' S and 67° 57' W) situated in the northeast of Isla Grande de Tierra del Fuego. They were recovered from the surface of an aeolian deposit, over a low hill 3700 m from the Atlantic coast. The remains did not show an associated context; they belong to an adult male and include only 2% of the skeleton.

Río Grande (Argentina): 530-320 BP

- I12354: 530-320 cal BP (520±30 BP, PSUAMS-6672)

The Río Grande is a river located in the northeast of Isla Grande de Tierra del Fuego. The city of Río Grande, Argentina, is located at the river mouth. One sample (I12354) analyzed in this study is derived from an adult male who was found near this river. He is registered in the Museo del Fin del Mundo under identifier 2410. He comes from a private donation and there is no exact information about his context. The approximate location is 53° 47' S and 67° 42' W.

Margen Sur (Argentina): 910-650 BP

- I12364: 910-670 cal BP (897±38 BP, AA69655)
- I12365: 800-680 cal BP (960±20 BP, PSUAMS-6684)
- I12367: 750-650 cal BP (895±25 BP, PSUAMS-6686)

The Margen Sur site (53° 49' 05'' S 67° 39' 22'' W) is located at approximately 1500 m from the Atlantic coast, close to the littoral beach ridge plain developed between Cabo Peñas and Punta Popper[27], in the northeast of Isla Grande de Tierra del Fuego. A deposit of aeolian sediments contains a shell midden intercepted by a primary multiple human burial composed by four subadult individuals. All the skeletons were lying in an extended position. Individuals B, C, and D were analyzed in this work.

One sample (I12364) analyzed in this study is derived from individual B, a subadult of 6-8 years old. Its skeletal completeness is 57% and it presents a bone injury, an ankylosis of the 2nd and 3rd cervical vertebrae, which was probably a congenital condition. Stable isotope values of $\delta^{13}C(col)= -19.9‰$, and $\delta^{15}N= 10.97‰$ obtained for this individual indicate a diet based predominantly on terrestrial resources. The second sample (I12365) came from individual D, a subadult of 4-8 years old with an integrity of 61.5%. Stable isotope values of $\delta^{13}C(col)= -18.29‰$, and $\delta^{15}N= 13.60‰$ were obtained for

this individual from prior studies[16,28], while in this study the values $\delta^{13}C(col)= -18.13‰$ and $\delta^{15}N= 15.10‰$ were obtained, both suggesting a terrestrial-based diet. The third sample (I12367) is from individual C, a subadult of 8-11 years old. Its skeletal completeness is 61.5%. Stable isotope values of $\delta^{13}C(col)= -18.35‰$, and $\delta^{15}N= 13.04‰$ were obtained from prior studies[16,28], while in this study the values $\delta^{13}C(col)= -17.92‰$ and $\delta^{15}N= 13.78‰$ were obtained, which concur in indicating a diet based on terrestrial resources[16,28].

A radiocarbon dating performed on *Mytilus chilensis* shells from the shell midden yielded 1295±50 BP (AA69657[29]).

<u>Río Policarpo (Argentina):</u> 500-320 BP

- I12361: 500-320 cal BP (815±20 BP, PSUAMS-6677)

The Río Policarpo is a river situated in the east of Isla Grande de Tierra del Fuego in the Mitre Peninsula. It drains into the Atlantic Ocean about 5 kilometers west from Caleta Falsa. One sample (I12361) analyzed in this study is derived from a subadult (5-7 years old) who was found near this river. He is registered in the Museo del Fin del Mundo under identifier 2673. He comes from a private donation and there is no exact information about its context. The approximate location is: 54° 41' S 65° 31' W.

<u>Caleta Falsa (Argentina):</u> 490-130BP

- I12356: 500-130 cal BP (650±20 BP, PSUAMS-6674)
- I12357: 480-140 cal BP (530±45 BP, PSUAMS-6675)
- I12358: 620-300 cal BP (820±40 BP, UGAMS-5151)
- I12359: 670-510 cal BP (870±20 BP, PSUAMS-6683)
- I12360: 490-310 cal BP (800±20 BP, PSUAMS-6676)

Caleta Falsa is an archaeological site in the north of the Mitre Peninsula at the eastern end of Isla Grande de Tierra del Fuego; it receives its name from a small bay. It was prospected and excavated by Anne Chapman, who worked in the area in 1970. A total of eight sites were defined. In general, they yielded lithic and bone artifacts, and the bones of guanaco, birds, seal, and whale. Almost without exception, the chipped stone artifacts are made of coarse-grained, gray to dark gray chert and quartzites obtained from cobbles along the Atlantic shore. It is assumed that all the sites are relatively recent, representing Haush occupations within the last several hundred years, based on the fact that typical materials of early occupations in the Tierra del Fuego region are not represented in the collection[30]. In three of the sites (3, 7, and 8), human remains corresponding to six individuals were recovered. All of

them were found in an articulated position, with a good state of preservation, since they were found in sub-superficial contexts. An exhaustive analysis of paleopathological lesions can be found in Guichón and Suby (2011)[13].

Site 3 is situated at Cabo Lata (or Donata), about 7 kilometers West from Caleta Falsa. (approximate location 54° 39' S 65° 33' W). It consists of an extensive workshop, or perhaps a series of small overlapping camping-workshop sites that were eroded by wind in large sand dunes along the coast[30].

One sample (I12356) analyzed in this study is from an incomplete subadult individual (13-17 years old) from this site. It is represented only by the skull and jaw. Stable isotope values of $\delta^{13}C$(col)= −11.6‰, $\delta^{13}C$(ap)= −9.9‰, and $\delta^{15}N$= 18.3‰ were obtained in a prior study[31], while in our study values were $\delta^{13}C$(col)= −10.55‰ and $\delta^{15}N$= 19.90‰, both consistent with a diet with a predominance of consumption of marine resources. The remains of this individual are kept in the Museo del Fin del Mundo where they are registered under identifier S3-3.

Site 7 is a shell midden located on the north side of Caleta Falsa. The principal midden accumulation is about 20 m from the beach, with the central part of the midden roughly 6 m in maximum diameter (north-south), 5 m in minimum diameter and 1.30 m high[30]. The approximate location is 54° 38' S 65° 27' W. A shell layer was encountered at 10-15 cm below the surface and was about 60 cm thick. Underlying this, there was a charcoal-stained zone about 30 cm in thickness. Here, two adult female individuals were buried[13].

One sample (I12357) analyzed in this study is derived from a female adult individual (30-44 years old) from this site. It was dorsally extended, with the skull directed south and facing east. A finely-made expanding stem arrow point of tan-gray fine-grained chert was found with the burial[30]. A mild tabular erect cranial deformation was observed[13]. Stable isotope values of $\delta^{13}C$(col)= −11.8‰, $\delta^{13}C$(ap)= −9.7‰, and $\delta^{15}N$= 18.5‰ were obtained in a prior study[31], while in our study values of $\delta^{13}C$(col)= −17.26‰ and $\delta^{15}N$= 13.05‰ were obtained, both in agreement with a diet with a predominance of consumption of marine resources. Now, the remains of this individual are kept in the Museo del Fin del Mundo where they are registered under the code S7-2.

Site 8 is situated near Site 7, on the northwest side of Caleta Falsa on the edge of a cliff. The approximate location is 54° 38' S 65° 27' W. Chapman carried out exploratory test excavations in various parts of the site, revealing a midden deposit composed of marine shells, bones of guanaco, whale, fish, birds, and seal, as well as quantities of lithic debris. During the course of these excavations, Chapman found two burials. One was that of an adult in an extended position. The other was a double interment consisting of an adult in a flexed position and the upper portions of a child's skeleton lying nearby[30].

One sample (I12358) analyzed in this study is from an adult male (18-23 years old) from this site. It is a simple burial that was found 30 m from the top of the cliff and 50 cm depth, the last 10 to 15 cm of which were formed by sterile earth and the rest with an anthropic origin, almost entirely covered by a limestone slab. The skeleton was almost complete, in an extended dorsal decubitus position, with the head to the north and the face upwards. It is a primary burial and it was not accompanied by artifacts. Some faunal remains also came from this same site. A stable isotope value of $\delta^{13}C(col) = -12.3‰$ was obtained in association with the date[13]. This value is consistent with a diet with a predominant consumption of marine resources. The remains of this individual are kept in the Museo del Fin del Mundo where they are registered under identifier S8-1.

The other burial of this site was double and was on the same slope of the ravine, also at 50 cm of the surface, under 20 cm of sterile earth and 30 cm of anthropogenic origin. The two skeletons were spread out on hard sterile sediment. A large amount of charcoal was found and it was submitted for radiocarbon dating, giving an age of 850±70 BP (UCLA-1912[30]).

Sample I12359 is derived from a male adult (43-49 years old) from this site. He presents 53% skeletal completeness. He was in a flexed position on the pelvis, on the left side, head to the northeast, side face facing east, right arm flexed at the elbow, hand on the lower part of the body, the other arm extended along the body and both legs bent towards the northwest, in the opposite direction to the face[13]. Stable isotope values of $\delta^{13}C(col) = -11.3‰$, $\delta^{13}C(ap) = -10.6‰$, and $\delta^{15}N = 15.1‰$ were obtained in a prior study[31], while in this study values of $\delta^{13}C(col) = -11.40‰$ and $\delta^{15}N = 18.47‰$ were obtained, both consistent with a diet with a predominant consumption of marine resources. Now, the remains of this individual are kept in the Museo del Fin del Mundo where they are registered under identifier S8-4.

Sample I12360 analyzed in this study is derived from the other individual of the double burial, a subadult of 2.5-5 years old. The elements of the lower extremities were not recovered, possibly because they were displaced during the burial of the adult male. If so, it would represent a previous burial in the same site. Its position was extended decubitus dorsal, face up looking to the east, at an angle of about 45º below the other skeleton. The skull was fractured postmortem, partially flattened on the sides. Above the skull, there was an artifact of polished bone (possibly a scapula of a whale), with one tip pointed and the other curved and measuring 116 mm long, 31 mm wide and 4 mm thick[13]. Now, the remains of this individual are kept in the Museo del Fin del Mundo where they are registered under identifier S8-5.

<u>Río Pipo (Argentina):</u> 1690-1350 BP

- I12355: 1690-1350 cal BP (1975±25 BP, PSUAMS-6673)

Río Pipo is a river in the south of Isla Grande de Tierra del Fuego that drains into the Beagle Channel. One sample (I12355) analyzed in this study is derived from an adult male (20-34 years old) who was found near this river. He is registered in the Museo del Fin del Mundo under the identifier number 2669. He comes from a private donation and there is no precise information about its context. His approximate location is 54° 51' S 68° 21' 12" W. A stable isotope value of $\delta^{13}C$(col)= −13.1‰ was obtained[32]. Long bones of this individual were analyzed by Tafuri *et al.*, 2017[33] and produced stable isotope values of $\delta^{13}C$(col)= −13.3‰ and $\delta^{15}N$= 15.8‰, while the values obtained via teeth in this study were $\delta^{13}C$(col)= −11.76‰ and $\delta^{15}N$= 19.06‰. These are consistent with a mixed diet with a slight predominance of consumption of marine resources.

<u>Almanza (Argentina):</u> 400-0 BP

- I12941: 390-0 cal BP (555±20 BP, PSUAMS-6678)
- I12943: 400-0 cal BP (570±20 BP, PSUAMS-6680)

Almanza is a river located at the south of Isla Grande de Tierra del Fuego, which drains into the Beagle Channel. A small port is located in the river mouth, about 75 kilometers east of the city of Ushuaia (approximate location 54º 47' S 68º 14' W). In 1975, during the construction of a route near this port, the remains of two individuals were found. They were deposited in the Museo del Fin del Mundo and both exhibit very good preservation and integrity.

One sample (I12941) analyzed in this study is derived from an adult male (40-45 years old). He is registered in the Museum with identifier 796. There is no more information about this sample.

The other sample (I12943) is derived from an adult male (35-40 years old). He is registered in the Museum under identifier 795. Stable isotope value of $\delta^{13}C$(col)= −12.1‰ was obtained by Tessone *et al.*, 2003[32]. This individual was also analyzed for stable isotope values by Tafuri *et al.*, 2017[33], producing values of $\delta^{13}C$(col)= −11.9‰ and $\delta^{15}N$= 18.0‰ (the values obtained in this study were $\delta^{13}C$(col)= −11.43‰ and $\delta^{15}N$= 19.12‰), consistent with a diet with a predominance of marine resources. One sample from this individual was previously analyzed for mitochondrial DNA and belongs to haplogroup C (Sample #F35 in García-Bour *et al.*, 2004[34]).

<u>Acatushún (Argentina):</u> 140-0 BP

- I12942: 140-0 cal BP (490±25 BP, PSUAMS-6679)

Acatushún is a shell midden located near the main house of Harberton farm at the South of Isla Grande de Tierra del Fuego. It is 40 meters from the shore of the Beagle Channel and 4 meters above sea level (54° 52.4' S 67° 19.91' W). In 1972, a group of amateurs dug up the remains of three individuals, two subadults and a perinate. They were donated to the Museo del Fin del Mundo. One sample (I12942) analyzed in this study is from a subadult registered in the Museum with identifier 855.

Supplementary Note 3. Spanish translation of abstract

Los registros arqueológicos documentan importantes cambios tecnológicos entre las poblaciones que vivieron en el extremo sur de Sudamérica (Patagonia Austral) durante los últimos trece milenios, tales como el desarrollo de las economías marinas y cambios en herramientas y en materias primas. Se ha propuesto que el movimiento de personas esparciendo cultura y tecnología impulsó algunos de estos cambios, pero estas hipótesis no han sido puestas a prueba con estudios de ADN antiguo. En este trabajo reportamos datos del genoma de 20 individuos antiguos, que se co-analizaron con datos previamente reportados. Por un lado, revelamos que la migración no explica el surgimiento de la adaptación marina en Patagonia Austral. A su vez, describimos continuidad genética parcial desde aproximadamente 6600 años AP y dos eventos de flujo génico posteriores correlacionados con cambios tecnológicos: uno entre 4700-2000 años AP que afectó principalmente a grupos marítimos, y un evento posterior que tuvo impacto en todos los grupos de la Patagonia Austral <2000 años AP. Finalmente, hace alrededor de entre 2200 y 1200 años AP, la mezcla entre poblaciones vecinas resultó en una clina correlacionada con el ordenamiento geográfico a lo largo de la costa.

Supplementary Note 4. Description of main findings in Spanish

Una mirada genética sobre la historia de las poblaciones de Patagonia Austral

En este estudio encontramos que dos de los individuos más antiguos de la región estudiados hasta el momento (que tienen una antigüedad de entre 5800 y 6600 años) son muy similares genéticamente a pesar de estar asociados a distintas estrategias de aprovechamiento de los recursos. Uno fue hallado en la costa del Estrecho de Magallanes en la península de Brunswick (Chile) y está asociado al consumo de recursos marítimos y el otro fue hallado en el norte de la Isla Grande de Tierra del Fuego (Argentina) y está asociado al consumo de recursos terrestres. Este hallazgo nos hace pensar que las distintas estrategias de adaptación no están asociadas a poblaciones diferentes, sino que fue una

misma población que llegó a la región y que luego implementó distintas maneras de aprovechar los recursos, según la zona donde se ubicó.

Por otra parte, hallamos que los individuos del Canal de Beagle, del norte de Tierra del Fuego y del sur del continente, de entre 2000 y 100 años de antigüedad, son descendientes de esos individuos más antiguos, pero también descienden de nuevos pobladores que llegaron posteriormente a la región. Por el contrario, en los individuos hallados en los Archipiélagos Occidentales (Chile) no se detecta la señal genética de los individuos más antiguos, sino que éstos descienden únicamente de poblaciones que llegaron más tardíamente desde el norte siguiendo la costa del Pacífico.

Según los datos analizados, en los individuos de los últimos dos milenios, se detectan dos migraciones independientes. Una que habría llegado por la vertiente pacífica hace entre 4700 y 2000 años, asociada a los grupos canoeros y que se encuentra representada en los grupos de los Archipiélagos Occidentales y del Canal de Beagle. Y la otra que habría llegado alrededor de 2000 años antes del presente por la vertiente atlántica desde el centro de Chile y que se encuentra representada en todas las poblaciones de la región. Estas migraciones pueden asociarse, respectivamente, a ciertos cambios tecnológicos, tales como el abandono del uso de la obsidiana verde en los archipiélagos occidentales y la aparición de un nuevo tipo de punta de proyectil en el sur del continente y el norte de Tierra del Fuego.

En cuanto a las poblaciones de la península Mitre de los últimos 400 años, se encontró que las mismas son descendientes de poblaciones del norte de la Isla Grande de Tierra del Fuego y del Canal de Beagle. Este proceso de mestizaje habría ocurrido hace unos 1500 años aproximadamente. Y la combinación genética se corresponde con las características tecnológicas de las poblaciones de esta región, que incluyen similitudes con sus vecinos tanto del norte como del sur de la isla.

Se encontró también que entre los 2200 y 1200 años antes del presente, todos los grupos del sur de Patagonia experimentaron un proceso de mestizaje con sus vecinos más cercanos, lo cual generó similitudes genéticas entre los individuos que se correlacionan con la distancia a lo largo de la línea de costa.

Por último, en los casos en los que se cuenta con información genómica de representantes actuales de las comunidades originarias (Kaweskar y Yaghanes de Chile), se pudo determinar que existe una continuidad genética entre los individuos antiguos de los últimos dos milenios y los individuos que viven actualmente en el mismo territorio.

Supplementary Note 5. Description of Radiocarbon Dating Methodology

Radiocarbon dating of the 14 new individuals were done at Arizona [AA] (1), Pennsylvania State University [PSUAMS] (12), and Bern [B] (1). Supplementary Data 1 provides details of the individuals associated with each laboratory's procedure. In-depth details of the procedures are described in Olalde *et al.*, 2019[35] (for AA and PSUAMS) and Szidat *et al.*, 2017[36] (for B). A summary of the procedure is described below:

When collagen samples were too poorly preserved, they were pre-treated using a modified XAD process[37]. These samples were cleaned using hand tools and sectioned with disposable Dremel cut-off wheels and then demineralized in 0.5 N HCl for 2-3 days at 5°C. The demineralized collagen pseudomorph was then gelatinized at 60°C in 1-2 mL 0.01 N HCl for eight to ten hours. Sample gelatin was pipetted into a pre-cleaned 10 mL disposable syringe with an attached 0.45 mm Millex Durapore PVDF filter (pre-cleaned with methanol and Nanopure H2O) and put into a thick-walled culture tube. The filtered solution was lyophilized and the percent gelatinization and yield were determined by weight. The sample gelatin was then hydrolyzed in 2 mL 6 N HCl for 22 h at 110°C. Supelco ENVI-Chrom® SPE (Solid Phase Extraction; Sigma-Aldrich) columns were prepped with 2 washes of HCl (2 mL) and rinsed with 10 mL DI H2O. The SPE Column was equilibrated with 50 mL 6 N HCl with a 0.45 mm Millex Durapore filter attached, and the washings were discarded. 2 mL collagen hydrolyzate as HCl was pipetted onto the SPE column and driven with an additional 10 mL 6 N HCl dropwise with the syringe into a 20 mm culture tube. The hydrolyzate was finally dried into a viscous syrup by passing UHP N_2 gas over the sample heated at 50°C for ~12 h.

Carbon and nitrogen concentrations and stable isotope ratios of the XAD amino acid samples were measured with a Costech elemental analyzer (ECS 4010) and Thermo DeltaPlus analyzer[38]. Sample quality was evaluated by % crude gelatin yield, %C, %N and C/N ratios before AMS ^{14}C dating. Samples (~2.1 mg) were then combusted for 3 h at 900°C in vacuum-sealed quartz tubes with CuO and Ag wires. Sample CO_2 was reduced to graphite at 550°C using H_2 and a Fe catalyst, with reaction water drawn off with $Mg(ClO_4)_2$. Graphite samples were pressed into targets in Al boats and loaded on a target wheel with OX-1 (oxalic acid) standards, known-age bone secondaries, and a ^{14}C-free Pleistocene whale blank. Samples were cleaned to remove adhering residues, and then acid-etched to remove secondary carbonate prior to hydrolysis. After rinsing in Nanopure H_2O and drying at 50°C, samples were evaluated for the integrity of their enamel using Fourier Transform Infared Spectroscopy. Samples and standards were placed then in BD Vacutainer septum-stopper vials, and digested with 85% orthophosphoric acid. The evolved CO_2 was graphitized as above and the ^{14}C measurements were made on a modified National Electronics Corporation compact spectrometer with a 0.5 MV accelerator (NEC 1.5SDH-1).

Supplementary References

1 Tessone, A., Guichón, R. A., Suby, J. & Kozameh, L. Bioarqueología de Península Mitre. *Los cazadores-recolectores del extremo oriental fueguino. Arqueología de Península Mitre e Isla de los Estados*, 231-270 (2011).

2 Gusinde, M. *Los indios de Tierra del Fuego. Vol. 1 y 2. Centro Argentino de Etnología Americana, CONICET* (1982).

3 Saletta, M. J., Vázquez, M., Elkin, D. & Oría, J. Los Selk´nam y los Haush de Tierra del Fuego. *Patrimonio a orillas del mar. Arqueología del litoral atlántico de Tierra del Fuego. Editora Cultural Tierra del Fuego*, 103-118 (2017).

4 Viegas Barros, J. P. La familia lingüística tehuelche. *Revista Patagónica* **54**, 39-46 (1992).

5 Viegas Barros, J. P. Explorando la hipótesis de parentesco Alacalufe-Yagan. *Lengua y Literatura Mapuche* **6**, 281–285 (1994).

6 Piana, E. L. & Orquera, L. A. The southern top of the world: The first peopling of Patagonia and Tierra del Fuego and the cultural endurance of the Fuegian Sea-Nomads. *Arctic Anthropology* **46**, 103-117 (2009).

7 Hernández, M., García-Moro, C. & Lalueza-Fox, C. Stature estimation in extinct Aónikenk and the myth of Patagonian gigantism. *American Journal of Physical Anthropology* **105**, 545-551 (1998).

8 Cocilovo, J. A., Varela, H. H. & Valdano, S. G. Hurgando en el arcón de los tiempos perdidos en los confines de la ecúmene. Las etnias selk'nam, yámana y halakwulup según el registro somatométrico de Martín Gusinde (1989 [1937]). *Intersecciones en Antropología* **20**, 69-78 (2019).

9 Guichón, R. A., Marti, I., Aspillaga, E. & Rothhammer, F. Contribución al conocimiento de las relaciones biológicas entre las poblaciones aborígenes de Patagonia Austral y Tierra del Fuegos. *RUNA* **19**, 27-39 (1990).

10 Hernández, M., Fox, C. L. & Garcia-Moro, C. Fueguian cranial morphology: the adaptation to a cold, harsh environment. *American Journal of Physical Anthropology* **103**, 103-117 (1997).

11 Hernández, M. Morfología craneal de las etnias de la Tierra del Fuego: diferencias sexuales e intergrupales. *Anales del Instituto de la Patagonia, Ciencias Humanas* **21**, 81-98 (1992).

12 González-José, R., Martínez-Abadías, N., Van Der Molen, S., García-Moro, C., Dahinten, S., & Hernandéz, M. (2004). Hipótesis acerca del poblamiento de Tierra del Fuego-Patagonia a partir del análisis genético-poblacional de la variación craneofacial. *Magallania (Punta Arenas)* **32**, 79-98.

13 Guichón, R. A. & Suby, J. A. Estudio bioarqueológico de los restos óseos humanos recuperados por Anne Chapman (T) en Caleta Falsa, Tierra del Fuego. *Magallania (Punta Arenas)* **39**, 163-177 (2011).

14 Guichón, R., Suby, J. & Fugassa, M. El Registro Biológico Humano en PatagoniaAustral. Algunas líneas de análisis. *Arqueología de la Costa Patagónica. Perspectivas para su conservación. Universidad Nacional de la Patagonia Austral y Secretaría de Cultura de la Provincia de Chubut*, 232-248 (2008).

15 Lenti, F. *Modello di catalogazione per la gestione di resti umani in Patagonia Australe. Università degli Studi di Cagliari, Facoltà di Scienze Matematiche, Fisiche e Naturali, Unpublished doctoral thesis* (2011).

16 Santiago, F., Salemme, M., Suby, J. & Guichón, R. Restos humanos en el norte de Tierra del Fuego: aspectos contextuales, dietarios y paleopatológicos. *Intersecciones en Antropología* **12**, 147-162 (2011).

17 Contreras, Á. B. Teoría y práctica de la arqueología de rescate. *Boletín de Arqueología de la Fian* **5**, 41-54 (1990).

18 Caracotche, M. S. & Ladrón De Guevara, B. El registro arqueológico costero de Patagonia: Diagnóstico del estado actual y herramientas para la conservación. *Arqueología de la Costa Patagónica. Perspectivas para la conservación. Universidad Nacional de la Patagonia Austral y Secretaría de Cultura de la Provincia de Chubut*, 17-50 (2008).

19 Scabuzzo, C., Frontini, R., Vecchi, R. & Bayón, C. Isótopos estables y dieta de los cazadores recolectores del sudoeste bonaerense (Argentina). *Chungará (Arica)* **48**, 383-395 (2016).

20 Martinic Beros, M. Hallazgo y excavación de una tumba Aonikenk en Cerro Johnny ("Brazo Norte") Magallanes. *Anales del Instituto de la Patagonia* **7**, 95-98 (1976).

21 Constantinescu, F. Evidencias bioantropológicas para modos de vida cazador recolector terrestre y marítimo en los restos óseos humanos de Tierra del Fuego. *Anales del Instituto de la Patagonia* **27**, 137-174 (1999).

22 Morello, F. *et al.* Punta Baxa 7: Sitio arqueológico de la costa norte de Tierra del Fuego, Estrecho de Magallanes (Chile). *Magallania (Punta Arenas)* **43**, 167-188 (2015).

23 Salemme, M., Santiago, F., Suby, J. & Guichón, R. Arqueología funeraria en el norte de Tierra del Fuego. *XVI Congreso Nacional de Arqueología Argentina. Jujuy* (2007).

24 Suby, J., Guichón, R., Salemme, M. & Santiago, F. Possibilities and limitations of human bone record in Southern Patagonia. *Fundhamentos* **7**, 349-363 (2008).

25 Salemme, M., Bujalesky, G. & Santiago, F. La Arcillosa 2: la ocupación humana durante el Holoceno medio en el Río Chico, Tierra del Fuego, Argentina. *Arqueología de Fuego-Patagonia. Levantando piedras, desenterrando huesos... y develando arcanos*, 723-736 (2007).

26 Suby, J. *Propiedades estructurales de restos humanos y Paleopatología en Patagonia Austral. Universidad Nacional de Mar del Plata. Unpublishe*d doctoral thesis (2007).

27 Bujalesky, G. G. Coastal geomorphology and evolution of Tierra del Fuego (Southern Argentina). *Geologica Acta: an international earth science journal* **5**, 337-362 (2007).

28 Zangrando, A. *et al.* Isótopos estables y dietas humanas en ambientes costeros. *Avances en arqueometría*, 91-97 (2004).

29 Salemme, M. B., M, Bártoli, V & Santiago F. La arqueofauna del sitio Margen Sur, Río Grande, Tierra del Fuego (Argentina). *IV Encuentro Latinoamericano de Arqueozoología, Libro de Resúmenes*, 160-170 (2018).

30 Chapman, A. & Hester, T. R. New data on the Archaeology of the Haush, Tierra del Fuego. *Journal de la Société des Américanistes* **62**, 185-208 (1973).

31 Yesner, D., Figuerero, M., Guichón, R. & Borrero, L. Análisis de isótopos estables en esqueletos humanos: confirmación de patrones de subsistencia etnográficos para Tierra del Fuego. *Shincal* **3**, 182-191 (1991).

32 Tessone, A., Zangrando, F. A., Valencio, S. & Panarello, H. Isótopos estables del carbono en restos óseos humanos de la región del Canal Beagle, Isla Grande de Tierra del Fuego. *Revista Argentina de Antropología Biológica* **5**, 33-43 (2003).

33 Tafuri, M. A. *et al.* Dietary resilience among hunter-gatherers of Tierra del Fuego: Isotopic evidence in a diachronic perspective. *PLoS ONE* **12**, e0175594 (2017).

34 García-Bour, J. *et al.* Early population differentiation in extinct aborigines from Tierra del Fuego-Patagonia: ancient mtDNA sequences and Y-chromosome STR characterization. *American Journal of Physical Anthropology* **123**, 361-370 (2004).

35 Olalde, I. *et al.* The genomic history of the Iberian Peninsula over the past 8000 years. *Science* **363**, 1230-1234 (2019).

36 Szidat, S., Vogel, E., Gubler, R. & Lösch, S. Radiocarbon dating of bones at the LARA Laboratory in Bern, Switzerland. *Radiocarbon* **59**, 831-842 (2017).

37 Lohse, J. C., Madsen, D. B., Culleton, B. J. & Kennett, D. J. Isotope paleoecology of episodic mid-to-late Holocene bison population expansions in the Southern Plains, USA. *Quaternary Science Reviews* **102**, 14-26 (2014).

38 Kennett, D. J. *et al.* Archaeogenomic evidence reveals prehistoric matrilineal dynasty. *Nature Communications* **8**, 14115 (2017).
