# Supplementary material for: Ancient genomes in South Patagonia reveal population movements associated with technological shifts and geography
Source: Nat Commun. 2020 Aug 3;11:3868. doi: 10.1038/s41467-020-17656-w (PMC7400565; doi:10.1038/s41467-020-17656-w)
Supplement: Supplementary file 3 — Descriptions of Additional Supplementary Files [file 41467_2020_17656_MOESM3_ESM.pdf]

Descriptions of Additional Supplementary Files

Supplementary Data 1

Description: Studied samples in this article. (A) Meta-data. (B) Dating correction details.

Supplementary Data 2

Description: Derived and ancestral allele counts (A,D) per individual for a set of variants of interest. DP: number of base calls aligned to a position.

Supplementary Data 3

Description: f3, f4 and FST results. Statistics of the form: (A) f4(Mbuti, X; MHoutPat, MHinPat) where X are individuals of interest, MHoutPat are individuals outside of Patagonia from the Middle Holocene and MHinPat is an individual within Patagonia from the Middle Holocene. (B) f4(Mbuti, X; MHinPat1, MHinPat2) where MHinPat1 and MHinPat2 are each an individual within Patagonia from the Middle Holocene and X are American individuals from different periods. (C) f4 (Mbuti, MHinPat; LH1, LH2) where LH1 and LH2 are individuals from the Late Holocene within Patagonia and MHinPat is an individual within Patagonia from the Middle Holocene. (D) f4(Mbuti, X; MHinPat, LHinPat) where LHinPat are Late Holocene Patagonians, MHinPat is an individual within Patagonia from the Middle Holocene and X are American individuals from different periods. (E) f4(Mbuti, Pat; Ch_MA, Ch_LIP) where Ch_MA and Ch_LIP are Chile_LosRieles_5100BP_MA and Chile_Conchali_700BP_LIP, respectively, and Pat are all Patagonian individuals. (F) f4(Mbuti, LHoutPat; LH1, LH2) where LH1 and LH2 are individuals from the Late Holocene within Patagonia and LHoutPat are individuals outside of Patagonia from the Late Holocene. (G) f4(Mbuti, ancY; X, Mixe) where ancY are modern Papuan individuals, Mixe are modern individuals from Mixe population in Mexico and X are ancient samples of interest in Patagonia. (H) f4(Mbuti, LH1; LH2, LH3) where LH1, LH2 and LH3 are individuals from the Late Holocene within Patagonia. (I) f3(LH1; LH2, LH3) where LH1, LH2 and LH3 are individuals from the Late Holocene within Patagonia. (J) f4(Mbuti, Modern; WA800BP, BC800BP) where Modern are modern individuals of interest, WA800BP are the two Western Archipelago samples from ~800BP (IPK13a and IPK12) and BC800BP are the two Beagle Channel from ~800BP (IPY08b and IPY10). (K) FST between pairs of Patagonian Late Holocene groups. For all f-statistics, standard errors were determined by jackknife resampling.

Supplementary Data 4

Description: Association tests between genetic distances and geographical and temporal distances, as well as distances based on diet and language. (A-B) Two-sided Spearman correlation, linear regression, and Mantel tests were performed when using 1-f3(Mbuti; Ind1, Ind2) and 1/f3(Mbuti; Ind1, Ind2) as genetic distances between two individuals, respectively.

Supplementary Data 5

Description: Estimated distances. (A) Geographical distances among sites, (B) Linguistic distances between geographical groups, (C) Diet distances between geographical groups, and (D) Temporal distances between samples.

Supplementary Data 6

Description: Results for qpAdm and DATES analyses. (A) qpAdm: Formal modeling of the Late Holocene individuals as mixes of the Early and Middle Holocene individuals and Chile_Conchali_700BP. (B) qpAdm: estimates of the proportions of ancestry in the different Late Holocene individuals as mixes of each other. Standard errors for qpADM were determined by jackknife resampling, accounting for the correlations between neighboring alleles and related populations, and p-values for ancestry waves were calculated by likelihood ratio two-sided tests. Thresholds for p-values were chosen to correct for multiple testing. (C) DATES: Estimates of the average time of admixture between two source populations to produce the target. Standard errors for DATES were determined by jackknife resampling.

Supplementary Data 7

Description: Copies of the Argentinean and Chilean governmental permissions to export the samples.
