# Supplementary material for: Ancient genomes in South Patagonia reveal population movements associated with technological shifts and geography
Source: Nat Commun. 2020 Aug 3;11:3868. doi: 10.1038/s41467-020-17656-w (PMC7400565; doi:10.1038/s41467-020-17656-w)
Supplement: Supplementary file 10 — Supplementary Data 7 [file 41467_2020_17656_MOESM10_ESM.pdf]

República Argentina - Poder Ejecutivo Nacional
2017 - Año de las Energías Renovables

Disposición

Número: DI-2017-50-APN-INAPL#MC

CIUDAD DE BUENOS AIRES
Miércoles 1 de Noviembre de 2017

Referencia: Exportación/Importación de Bienes Arqueológicos

VISTA la solicitud IF-2017-24071118-APN-INAPL#MC, del EX-2017-24071103-APN-INAPL#MC, efectuada por la Dra. Josefina María Brenda MOTTI para la exportación definitiva a Estados Unidos de seis (6) piezas dentales humanas procedentes de los sitios arqueológicos La Arcillosa 2, Margen Sur, Caleta Falsa y Pozo Tierra del Fuego 1 (Provincia de Tierra del Fuego e Islas del Atlántico Sur) y de dos (2) piezas dentales humanas procedentes del sitio Mar Chiquita (Provincia de Buenos Aires), a fin de realizar análisis especializados para la obtención de mitogenomas por técnica de Next-generation Sequencing, y

CONSIDERANDO:

Que el análisis será llevado a cabo en el Molecular Anthropology Laboratories Department of Anthropology University of Tennessee (Estados Unidos).

Que las muestras serán enviadas a destino mediante la empresa de correo postal Correo Argentino.

Que las muestras son analizadas en el marco del proyecto: “PIP 2015-2017 N° 11220150100953CO y PICT 2010 Nª 0575”.

Que la exportación de las muestras de los sitios procedentes de Tierra del Fuego cuenta con la autorización de la Dirección Provincial de Museos y Patrimonio Cultural-Secretaría de Cultura- de la Provincia de Tierra del Fuego e Islas del Atlántico Sur.

Que la exportación de la muestra del sitio de la Provincia de Buenos Aires cuentan con la autorización de la Dirección Provincial de Museos y Preservación Provincial de la Provincia de Buenos Aires (Nota N° 653).

Que en virtud de lo establecido en los artículos 4º y 5º de la Ley Nacional Nº 25.743 sobre “Protección del Patrimonio Arqueológico y Paleontológico” y de los términos de la Resolución S.C. Nº 2272 de fecha 29 de julio de 2004, referente a exportaciones de bienes arqueológicos.

Por ello:

LA DIRECTORA

DEL INSTITUTO NACIONAL DE ANTROPOLOGÍA Y PENSAMIENTO

LATINOAMERICANO

DISPONE:

ARTÍCULO 1º.- Autorizar a la Dra. Josefina María Brenda MOTTI para la exportación definitiva a Estados Unidos de seis (6) piezas dentales humanas procedentes de los sitios arqueológicos La Arcillosa 2, Margen Sur, Caleta Falsa y Pozo Tierra del Fuego 1 (Tierra del Fuego) y de dos (2) piezas dentales humanas procedentes del sitio Mar Chiquita (Provincia de Buenos Aires), a fin de realizar análisis especializados para la obtención de mitogenomas por técnica de Next-generation Sequencing.-

ARTÍCULO 2º.- Regístrese, comuníquese a la interesada. Cumplido, archívese.-

Digitally signed by ACUÑA Maria Leonor Elena
Date: 2017.11.01 15:35:06 ART
Location: Ciudad Autónoma de Buenos Aires

Maria Leonor Elena Acuña
Representante Titular
Instituto Nacional de Antropología y Pensamiento Latinoamericano
Ministerio de Cultura

Digitally signed by GESTION DOCUMENTAL ELECTRONICA - GDE
DN: cn=GESTION DOCUMENTAL ELECTRONICA - GDE, c=AR, o=MINISTERIO DE MODERNIZACION, ou=SECRETARIA DE MODERNIZACION ADMINISTRATIVA, serialNumber=CUIT 30715117564
Date: 2017.11.01 15:35:22 -03'00'

República Argentina - Poder Ejecutivo Nacional
2017 - Año de las Energías Renovables

Disposición

Número: DI-2017-72-APN-INAPL#MC

CIUDAD DE BUENOS AIRES
Jueves 28 de Diciembre de 2017

Referencia: Exportación/Importación de Bienes Arqueológicos

VISTA la solicitud IF-2017-30053121-APN-INAPL#MC del EX-2017-30053131-APN-INAPL#MC, efectuada por la Dra. Josefina María Brenda MOTTI para la exportación definitiva a Estados Unidos de siete (7) piezas dentales humanas procedentes de los sitios arqueológicos El Guanaco 1 y Laguna Toro (Provincia de Buenos Aires) y de once (11) piezas dentales humanas procedentes de los sitios arqueológicos Caleta Falsa, Puesto Pescador, Acatushun, Almanza, Río Grande, Río Policarpo y Río Pipo (Provincia de Tierra del Fuego e Islas del Atlántico Sur), a fin de realizar análisis especializados para la obtención de mitogenomas por técnica de Next-generation Sequencing, y

CONSIDERANDO:

Que el análisis será llevado a cabo en el Molecular Anthropology Laboratories Department of Anthropology University of Tennessee (Estados Unidos).

Que las muestras serán enviadas a destino mediante la empresa de correo postal Correo Argentino.

Que las muestras son analizadas en el marco del proyecto: “PIP 2015-2017 N° 11220150100953CO y PICT 2010 N° 0575”.

Que la exportación de las muestras procedentes de sitios ubicados en la Provincia de Buenos Aires cuenta con la autorización de la Dirección Provincial de Museos y Preservación Provincial de la Provincia de Buenos Aires.

Que la exportación de las muestras de los sitios procedentes de Tierra del Fuego cuenta con la autorización de la Dirección Provincial de Museos y Patrimonio Cultural-Secretaría de Cultura- de la Provincia de Tierra del Fuego e Islas del Atlántico Sur.

Que en virtud de lo establecido en los artículos 4º y 5º de la Ley Nacional Nº 25.743 sobre “Protección del Patrimonio Arqueológico y Paleontológico” y de los términos de la Resolución S.C. Nº 2272 de fecha 29 de julio de 2004, referente a exportaciones de bienes arqueológicos.

Por ello:

LA DIRECTORA

DEL INSTITUTO NACIONAL DE ANTROPOLOGÍA Y PENSAMIENTO

LATINOAMERICANO

DISPONE:

ARTÍCULO 1º.- Autorizar a la Dra. Josefina María Brenda MOTTI para la exportación definitiva a Estados Unidos de siete (7) piezas dentales humanas procedentes de los sitios arqueológicos El Guanaco 1 y Laguna Toro (Provincia de Buenos Aires) y de once (11) piezas dentales humanas procedentes de los sitios arqueológicos Caleta Falsa, Puesto Pescador, Acatushun, Almanza, Río Grande, Río Policarpo y Río Pipo (Provincia de Tierra del Fuego e Islas del Atlántico Sur), a fin de realizar análisis especializados para la obtención de mitogenomas por técnica de Next-generation Sequencing.-

ARTÍCULO 2º.- Regístrese, comuníquese a la interesada. Cumplido, archívese.-

Digitally signed by ACUÑA Maria Leonor Elena
Date: 2017.12.28 15:47:59 ART
Location: Ciudad Autónoma de Buenos Aires

Maria Leonor Elena Acuña
Representante Titular
Instituto Nacional de Antropología y Pensamiento Latinoamericano
Ministerio de Cultura

Digitally signed by GESTION DOCUMENTAL ELECTRONICA - GDE
DN: cn=GESTION DOCUMENTAL ELECTRONICA - GDE, c=AR, o=MINISTERIO DE MODERNIZACION, ou=SECRETARIA DE MODERNIZACION ADMINISTRATIVA, serialNumber=CUIT 30715117564
Date: 2017.12.28 15:48:08 -03'00'

Servicio Nacional
del Patrimonio
Cultural

Ministerio de las Culturas
las Artes y el Patrimonio

CERTIFICADO

Certifico que el Centro de Estudios del Hombre Austral del Instituto de la Patagonia, Universidad de Magallanes, posee un Depósito de Colecciones Patrimoniales reconocido por el Consejo de Monumentos Nacionales de Chile como la única institución universitaria autorizada para albergar colecciones patrimoniales (históricas, arqueológicas y antropológicas) de la región de Magallanes.

Que el Dr. Alfredo Prieto fue su director entre los años 2010 a 2014 , época en que se exportaron por mano (2010), bajo su responsabilidad , muestras científicas sin valor comercial contenidas en ese depósito y que correspondían a muestras de individuos de los sitios Faro Méndez (Tierra del Fuego) 54888 donado a esa institución en 1998 y de Cerro Johnny (Magallanes) ingresado a la colección en 1976. Dichas muestras son parte de un estudio a publicarse prontamente.

PABLO QUERCIA MARTINIC
DIRECTOR REGIONAL
SERVICIO NACIONAL DEL PATRIMONIO CULTURAL
REGIÓN DE MAGALLANES Y DE LA ANTÁRTICA CHILENA

Punta Arenas, 16 de junio de 2020
