# Supplementary material for: Ancient genomes in South Patagonia reveal population movements associated with technological shifts and geography
Source: Nat Commun. 2020 Aug 3;11:3868. doi: 10.1038/s41467-020-17656-w (PMC7400565; doi:10.1038/s41467-020-17656-w)
Supplement: Supplementary file 11 — Reporting Summary [file 41467_2020_17656_MOESM11_ESM.pdf]

nature research

Corresponding author(s): Nathan Nakatsuka, Pierre Luisi, Rodrigo Nores, David Reich

Last updated by author(s): Jul 3, 2020

Reporting Summary

Nature Research wishes to improve the reproducibility of the work that we publish. This form provides structure for consistency and transparency in reporting. For further information on Nature Research policies, see Authors & Referees and the Editorial Policy Checklist .

Statistics

For all statistical analyses, confirm that the following items are present in the figure legend, table legend, main text, or Methods section.

n/a	Confirmed	
☐	☒	The exact sample size (*n*) for each experimental group/condition, given as a discrete number and unit of measurement
☒	☐	A statement on whether measurements were taken from distinct samples or whether the same sample was measured repeatedly
☐	☒	The statistical test(s) used AND whether they are one- or two-sided<br>*Only common tests should be described solely by name; describe more complex techniques in the Methods section.*
☐	☒	A description of all covariates tested
☐	☒	A description of any assumptions or corrections, such as tests of normality and adjustment for multiple comparisons
☐	☒	A full description of the statistical parameters including central tendency (e.g. means) or other basic estimates (e.g. regression coefficient) AND variation (e.g. standard deviation) or associated estimates of uncertainty (e.g. confidence intervals)
☐	☒	For null hypothesis testing, the test statistic (e.g. *F*, *t*, *r*) with confidence intervals, effect sizes, degrees of freedom and *P* value noted<br>*Give P values as exact values whenever suitable.*
☐	☒	For Bayesian analysis, information on the choice of priors and Markov chain Monte Carlo settings
☐	☒	For hierarchical and complex designs, identification of the appropriate level for tests and full reporting of outcomes
☐	☒	Estimates of effect sizes (e.g. Cohen's *d*, Pearson's *r*), indicating how they were calculated

Our web collection on statistics for biologists contains articles on many of the points above.

Software and code

Policy information about availability of computer code

Data collection: Illumina NextSeq500 v.2 was used to sequence the DNA.

Data analysis: SeqPrep version 1.2 (https://github.com/jstjohn/SeqPrep) or custom software (https://github.com/DReichLab/ADNA-Tools) was used to merge paired forward and reverse reads. BWA version 0.6.1 (bio-bwa.sourceforge.net) was used to align the reads to the hg19 human genome reference sequence. Duplicates were removed with Picard version 2.23.0 (http://broadinstitute.github.io/picard/). OxCal version 4.3 (https://c14.arch.ox.ac.uk/oxcal.html) was used to calculate 14C ages. ContamMix version 1.0-12 (https://github.com/DReichLab/ADNA-Tools) was used to measure mitochondrial contamination. ANGSD version 0.930 (https://github.com/ANGSD/angsd) was used to measure X-chromosome contamination. Autosomal contamination was measured with ContamLD version 1.0 (https://github.com/nathan-nakatsuka/ContamLD). yHaplo (https://github.com/23andMe/yhaplo) was used to determine Y chromosome haplotypes. Samtools version 1.10 (http://samtools.sourceforge.net/) was used to generate VCFs from BAM files and find genotype information. Haplogrep version 17 (http://haplogrep.uibk.ac.at/index.html) was used to attain mitochondrial haplogroup assignments. ADMIXTURE version 1.3.0 (https://www.genetics.ucla.edu/software/admixture/download.html) was used to do unsupervised clustering analysis. RFMIX version 2 (https://github.com/slowkoni/rfmix) was used to find local ancestry estimates. shapeIT2 version 2 (https://jmarchini.org/shapeit2/) was used for phasing. smartPCA in EIGENSOFT version 5.0 (https://github.com/DReichLab/EIG) was used to perform Principal Components Analysis and FST analyses. f-statistics, qpAdm, and qpGraph were calculated with ADMIXTOOLS version 6.0 (https://github.com/DReichLab/AdmixTools). DATES version 1510 (https://github.com/priyamoorjani/DATES) was used to estimate the time of admixture in ancient DNA samples. Mantel tests were done with the multi.Mantel function in R in the phytools version 0.6-60 package (https://cran.r-project.org/web/packages/phytools/index.html). The geor package version 1.8 in R (https://cran.r-project.org/web/packages/geoR/index.html) and the maps package version 3.3.0 (https://cran.r-project.org/web/packages/maps/index.html) were used to obtain geographical distance estimates and a map. The ggplot2 package version 3.3.2 (https://cran.r-project.org/web/packages/ggplot2/index.html) was used to plot the map, the ggrepel package version 0.8.2 (https://cran.r-project.org/web/packages/ggrepel/index.html) was used to label the map, the dplyr package version 1.00 (https://cran.r-project.org/web/packages/dplyr/index.html) was used to obtain accents on the map, Conditional Heterozygosity was done using POPSTATS September 26, 2018 version (https://github.com/pontussk/popstats). Yfitter version 0.3 (https://sourceforge.net/p/yfitter/wiki/Home/) was used to confirm Y-chromosome haplogroup mutations. PLINK2 version 2.0 alpha (https://www.cog-genomics.org/plink/2.0/) was used to determine correlation between

SNPs. Damage rates were calculated with PMDTools version 0.60 (https://github.com/pontussk/PMDtools). Kinship was determined using custom software based on mismatch rates described in Kennett et al., 2017 (available upon request but not yet ready for broader distribution). Scripts for making outgroup-f3 neighbor-joining trees and MDS plots as well as counting alleles are available at https://github.com/nathan-nakatsuka/Patagonia.

For manuscripts utilizing custom algorithms or software that are central to the research but not yet described in published literature, software must be made available to editors/reviewers. We strongly encourage code deposition in a community repository (e.g. GitHub). See the Nature Research guidelines for submitting code & software for further information.

Data

Policy information about availability of data

All manuscripts must include a data availability statement. This statement should provide the following information, where applicable:

- Accession codes, unique identifiers, or web links for publicly available datasets
- A list of figures that have associated raw data
- A description of any restrictions on data availability

All sequencing data are available from the European Nucleotide Archive, accession number PRJEB39010. Genotype data obtained by random sampling of sequences at approximately 1.24 million analyzed positions are available from the Reich Laboratory website at: https:// reich.hms.harvard.edu/datasets.

Field-specific reporting

Please select the one below that is the best fit for your research. If you are not sure, read the appropriate sections before making your selection.

☒ Life sciences ☐ Behavioural & social sciences ☐ Ecological, evolutionary & environmental sciences

For a reference copy of the document with all sections, see nature.com/documents/nr-reporting-summary-flat.pdf

Life sciences study design

All studies must disclose on these points even when the disclosure is negative.

Sample size	Sample size was not pre-determined beforehand and was based on availability of skeletal material and ability to generate DNA from the material.
Data exclusions	Individual I12365 was excluded from the analyses as he is a brother of I12367 and has lower coverage data (but the data from I12365 we provide publicly).
Replication	Analyses were done (where possible and relevant) on both the group and individual level to determine if the results were biased by single individuals in the group. The analyses were also done across multiple datasets and processing methods and they were consistent across these different datasets. Most of the analyses were deterministic, so replication was not performed in these cases. For ADMIXTURE, RFMIX, and shapeIT2, EM iterations were set to the recommended number of times (10, 2, 20) and the replicate with the highest likelihood was taken (for shapeIT2, the average of the main iterations was taken as recommended by the software developers). In all of these cases there was minimal difference between the iterations.
Randomization	No randomization of the individuals was done because the analyses were not based on experimental treatment of different groups looking forward in time. The analyses were based on comparisons of the genetics of the different groups (looking backwards in time) where knowledge of the groups is necessary to attain the results.
Blinding	Blinding was not relevant to this study for the same reason as stated above for randomization.

Reporting for specific materials, systems and methods

We require information from authors about some types of materials, experimental systems and methods used in many studies. Here, indicate whether each material, system or method listed is relevant to your study. If you are not sure if a list item applies to your research, read the appropriate section before selecting a response.

Materials & experimental systems

n/a	Involved in the study	
☒	☐	Antibodies
☒	☐	Eukaryotic cell lines
☐	☒	Palaeontology
☒	☐	Animals and other organisms
☒	☐	Human research participants
☒	☐	Clinical data

Methods

n/a	Involved in the study	
☒	☐	ChIP-seq
☒	☐	Flow cytometry
☒	☐	MRI-based neuroimaging

Palaeontology

Specimen provenance	All Argentina samples analyzed in this work were exported with authorization from the Instituto Nacional de Antropología y Pensamiento Latinoamericano (INAPL) N° DI-2017-50-APN-INAPL#MC dated November 1st, 2017 and N° DI-2017-72-APN-INAPL#MC dated December 28th, 2017. These permissions were given under the understanding that the export of samples from sites from Tierra del Fuego travel with the authorization of the Provincial Office of Museums and Cultural Heritage Ministry of Culture, province of Tierra del Fuego, Antarctica and South Atlantic islands, and this procedure was followed for the present study. The export of the sample from Laguna Toro, province of Buenos Aires, had the authorization from the government's Direction of Museums and Preservation of Buenos Aires Province. The Chilean individuals were sent with permissions from the Chilean National Monuments Council (CMN) in 2009.
Specimen deposition	The Argentina ancient individuals are all currently kept in the Museo del Fin del Mundo (MFM) (in the jurisdiction of the Provincial Office of Museums and Cultural Heritage - Ministry of Culture, province of Tierra del Fuego, Antarctica and South Atlantic islands, Argentina. The Chilean ancient individuals Universidad de Magallanes (Centro de Estudios del Hombre Austral, Instituto de la Patagonia, Chile.
Dating methods	We report 15 new direct AMS 14C bone dates of 14 ancient individuals from 2 radiocarbon laboratories (Bern, Switzerland [B] = 2 for a single individual, and Pennsylvania State University [PSUAMS] = 13) (Supplementary Online Table 1). Methods for each lab are the same as those used in the following publications, and we refer to those publications for the details: Bern: Szidat et al., 2017; PSUAMS: Olalde et al., 2019. The 14C ages were calculated using OxCal version 4.3 using differing mixtures of the southern hemisphere terrestrial (SHCal13) and the marine (Marine13) calibration curves.

☒ Tick this box to confirm that the raw and calibrated dates are available in the paper or in Supplementary Information.
